# Stochastically Gating Ion Channels Enable Patterned Spike Firing through Activity-Dependent Modulation of Spike Probability

**DOI:** 10.1371/journal.pcbi.1000290

**Published:** 2009-02-13

**Authors:** Joshua T. Dudman, Matthew F. Nolan

**Affiliations:** 1 Janelia Farm Research Campus, Howard Hughes Medical Institute, Ashburn, Virginia, United States of America; 2 Centre for Integrative Physiology, R(D)SVS, University of Edinburgh, Edinburgh, United Kingdom; UFR Biomédicale de l'Université René Descartes, France

## Abstract

The transformation of synaptic input into patterns of spike output is a fundamental operation that is determined by the particular complement of ion channels that a neuron expresses. Although it is well established that individual ion channel proteins make stochastic transitions between conducting and non-conducting states, most models of synaptic integration are deterministic, and relatively little is known about the functional consequences of interactions between stochastically gating ion channels. Here, we show that a model of stellate neurons from layer II of the medial entorhinal cortex implemented with either stochastic or deterministically gating ion channels can reproduce the resting membrane properties of stellate neurons, but only the stochastic version of the model can fully account for perithreshold membrane potential fluctuations and clustered patterns of spike output that are recorded from stellate neurons during depolarized states. We demonstrate that the stochastic model implements an example of a general mechanism for patterning of neuronal output through activity-dependent changes in the probability of spike firing. Unlike deterministic mechanisms that generate spike patterns through slow changes in the state of model parameters, this general stochastic mechanism does not require retention of information beyond the duration of a single spike and its associated afterhyperpolarization. Instead, clustered patterns of spikes emerge in the stochastic model of stellate neurons as a result of a transient increase in firing probability driven by activation of HCN channels during recovery from the spike afterhyperpolarization. Using this model, we infer conditions in which stochastic ion channel gating may influence firing patterns *in vivo* and predict consequences of modifications of HCN channel function for *in vivo* firing patterns.

## Author Summary

Neurons use electrical impulses called action potentials to transmit signals from their cell body to their axon terminals, where the impulses trigger release of neurotransmitter. Initiation of an action potential is determined by the balance of currents through ion channels in a neuron's membrane. Although it is well established that membrane ion channels randomly fluctuate between open and closed states, most models of action potentials account for the average current through these channels but not for the current fluctuations caused by this stochastic opening and closing. Here, we examine the consequences of stochastic ion channel gating for stellate neurons found in the entorhinal cortex. The intrinsic properties of these neurons cause characteristic clustered patterns of spiking. We find that in a model of a single stellate neuron that is constrained by previous experimental data clustered action potential patterns are produced only when the model accounts for the random opening and closing of individual ion channels. This stochastic model provides an example of a general mechanism for patterning of neuronal activity and may help to explain the patterns of spikes fired by entorhinal neurons that encode spatial location in behaving animals.

## Introduction

Thermal fluctuations in the conformation of an ion channel protein can cause it to make spontaneous transitions between discrete conducting and non-conducting states [1],[2]. Nevertheless, computational models of ionic conductances in a neuron generally assume the behavior of a population of ion channels to be deterministic and stochastic gating of ion channels is usually neglected in models of synaptic integration and spike initiation [3],[4]. For a typical cortical principal neuron, this assumption can be justified by the very small amplitude of the conductance change and resulting membrane current caused by opening of a single ion channel compared to either the resting membrane conductance or the threshold current for firing of an action potential. However, when neurons are depolarized to membrane potentials around the threshold for initiation of action potentials, the biophysical mechanisms that underlie spike generation dictate that the effective membrane conductance becomes very low [5]. As a result, even small fluctuations in ionic current through relatively few ion channels could significantly alter the membrane potential and the initiation of action potentials [6],[7]. Consistent with this possibility stochastic gating of membrane ion channels that determine the threshold for action potential initiation can influence the dynamic electrical properties of neurons [8]–[11]. However, little attention has been given to the consequences of stochastic ion channel gating for the patterns of spike output produced during active states in which the membrane potential is depolarized to near threshold.

We have focused on understanding the influence of stochastic ion channel gating on the integrative properties of stellate neurons from Layer II of the medial entorhinal cortex (MEC). These glutamatergic neurons provide cortical input to the hippocampal dentate gyrus [12],[13]. Electrophysiological recordings reveal two unusual integrative properties of stellate neurons from the MEC [14]–[17]. First, during prolonged periods of excitation stellate neurons fire action potentials in stereotypical clustered patterns. The frequency of spikes within a cluster is approximately 8–14 Hz and is relatively independent of the average spike frequency, while the intervals between spike clusters are typically hundreds of milliseconds or longer [18]. The organization of clustered spike patterns appears to depend on a large and slow spike afterhyperpolarization (AHP) that is also independent of the overall average spike frequency [18]. A second distinctive feature of stellate neurons is the emergence of prominent (∼3–5 mV in amplitude) intrinsic membrane potential fluctuations upon membrane depolarization [14]. These fluctuations have been proposed to contribute to network rhythmicity due to their power in the theta frequency range (4–12 Hz), the prominent oscillatory frequency of entorhinal and hippocampal network activity during exploratory behavior and REM sleep [19]. Previous models and experimental results indicate that stochastic gating of persistent Na^+^ channels may be essential for the sub-threshold oscillations observed in stellate neurons [20],[21]. However, the consequences of stochastic gating of other classes of ion channel expressed by stellate neurons have not been explored. Moreover, while sub-threshold oscillations have been suggested to drive clustered spike patterns [14],[22],[23], the mechanisms underlying oscillations and clustered spike firing can be dissociated experimentally [18],[24], and therefore it is not clear if stochastic fluctuations in ion channel opening play any role in the generation of clustered spike firing.

Hyperpolarization-activated, cation non-selective (HCN) channels play a central role in determining subthreshold integration and the pattern of action potential initiation in stellate neurons from the MEC [18],[25]. The substantial hyperpolarization-activated current (I_h_) in stellate cells is mediated in large part by HCN1 channels and is a major determinant of the effective membrane conductance of the neuron at rest and at more depolarized potentials close to the threshold for initiation of action potentials [18]. Experiments using pharmacological and genetic manipulations suggest that HCN channels increase the probability that clustered patterns of action potentials will be generated and increase the frequency of action potentials within each cluster [18]. However, the mechanisms through which HCN channels influence these patterns of spike firing are not clear. Computational models of stellate neurons have suggested either that I_h_ plays an essential role in perithreshold oscillations and clustered patterns of spike firing [26] or that I_h_ is not required [21]. Moreover, numerous studies suggest that the effects of I_h_ on the integrative properties of a neuron are highly context dependent [18], [27]–[36]. Thus, the role of I_h_ is determined by interactions with other ion channels. Depending upon the cell type and even the subcellular compartment studied, I_h_ can lead to varied properties, from prevention of bistability [37] to regulation of dendritic spiking [38]. Therefore, understanding the properties of stellate neurons and their sensitivity to manipulations of I_h_ will likely require an account of the interactions between multiples classes of ion channel.

To better understand the impact of stochastic ion channel gating on the patterns of spike output from stellate neurons and to reconcile the contrasting views of the role of I_h_ in perithreshold oscillations and clustered patterns of spike firing, we addressed two questions. How do interactions of HCN channels with other membrane ion channels lead to the emergence of membrane potential oscillations and spike firing patterns recorded from entorhinal stellate cells? Could stochastic ion channel gating at potentials close to spike threshold influence the patterns of spike output generated by stellate neurons? We demonstrate that whereas a deterministic model of channel gating is sufficient to account for many of the properties of entorhinal stellate neurons at hyperpolarized membrane potentials, including the consequences of HCN1 deletion, a model with stochastically gating ion channels is necessary to reproduce the distinctive properties of stellate neurons near threshold. Examination of the model reveals that spike initiation is probabilistic and that the tendency to emit clustered spikes can be explained by a transient increase in the probability of spike initiation following recovery from the action potential AHP. We find that this transient increase in spike probability is primarily due to I_h_ and explains the role of HCN channels in the emergence of clustered patterns of spikes. Finally, we ask whether stochastic ion channel gating could contribute to patterns of spike output observed *in vivo*. We propose that stochastic gating of ion channels expressed by stellate neurons is crucial to their transformation of synaptic input into a patterned spiking output and places constraints on the development of models of entorhinal cortex function [39].

## Results

To study the influence of stochastic gating of ion channels on the integrative properties of stellate neurons we implemented a single compartment model neuron endowed with ionic conductances derived from experimental data (see Materials and Methods). In the results sections that follow we first describe the key integrative properties of this model and show that they are similar to published experimental data. We then explore how clustered patterns of action potentials emerge in the model. Finally, to establish whether the model might explain firing patterns recorded from superficial entorhinal neurons in behaving animals, we simulate responses of the model to dynamic input.

**Figure 1:**
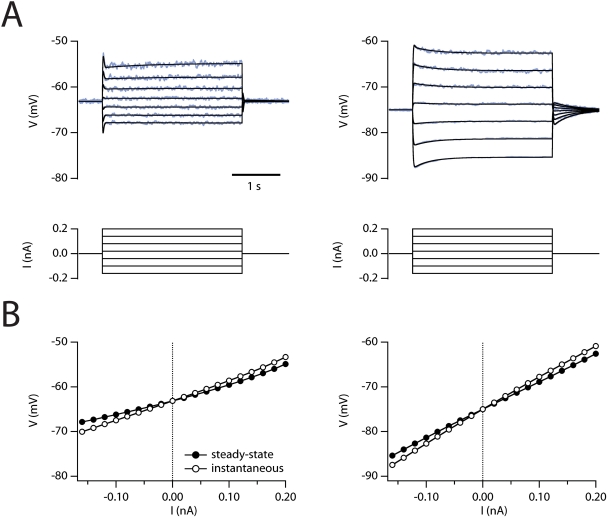
Subthreshold properties of the stellate neuron model. (A) Membrane potential responses (top) to current steps (bottom) are plotted for the wild-type (left) and HCN1 knockout (right) versions of the model. Traces in blue are from simulations with the stochastic models and traces in black from the deterministic version of the model. (B) Steady-state (closed symbols) and instantaneous (open symbols) voltage responses plotted as a function of current step amplitude for the wild-type (left) and HCN1 knockout (right) versions of the model.

Initially, we developed kinetic formalisms of the Hodgkin-Huxley type and solved for the resultant currents deterministically (Figure 1). Using the deterministic model we established that the single compartment model could account for the resting membrane properties of stellate neurons (Figure 1 and Table 1). As a further constraint we examined whether the model could account for previous experimental results in mice with global deletion of the gene encoding the HCN1 channel. Thus, in addition to a wild-type version of the model, we implemented a version in which the fast, large I_h_ was replaced by a smaller, slower current similar to that recorded in HCN1 knockout mice [18]. This single compartment, deterministic model replicated the basic effects of either HCN1 deletion or pharmacological blockade of I_h_ on the resting membrane properties of stellate cells (Figure 1 and Table 1).

**Table 1:** Passive membrane properties of the stellate neuron models.

	Wild-Type (Deterministic)	Wild-Type (Stochastic)	HCN1 Knockout (Deterministic)	HCN1 Knockout (Stochastic)
V_rest_ (mV)	−63.15	−63.13±0.07	−75.01	−74.97±0.05
R_i_+(MΩ)	33.5	34.2±1.1	61.7	59.3±1.7
R_i_−(MΩ)	32.2	32.3±1.4	62.1	61.4±1.5
τ_m_+(ms)	5.4	5.0±0.5	10.1	9.7±0.9
τ_m_−(ms)	5.4	5.6±0.9	10.1	9.8±1.0
Sag Ratio	0.73	0.72±0.08	0.83	0.84±0.03

Input resistance (R_i_) was defined as the ratio of the steady-state voltage change in response to positive (“+”) or negative (“−“) current injection from the resting membrane potential. Monoexponential fits to the initial voltage response were used to obtain the membrane time constant (τ_m_). The sag ratio is calculated as the ratio of the peak hyperpolarization divided by the steady-state hyperpolarization for the negative current injection. Parameter estimates from the stochastic models were determined from an average of 5 simulations. Errors are the standard deviation.

While previous studies have investigated the consequences of stochastic Na^+^ channel gating in models containing otherwise deterministic ion channels [21], as well as addition of a simulated stochastic Na^+^ conductance during experimental recordings from stellate neurons [20], models of stellate neurons in which all of the ion channels are stochastically gating have not been explored. To examine the effects of stochastic channel gating, all channels in both models were converted to first-order Markov models [1],[40],[41]. Consistent with previous studies [6],[8],[9], we find that even with the substantial channel densities that are required to match current amplitudes to values from whole-cell recordings, channel noise can cause significant deviations from the mean current (Figure S1). Nevertheless, the average resting membrane properties of the model are unaffected by the presence of stochastically rather than deterministically gating ion channels (Figure 1 and Table 1).

### Perithreshold Membrane Potential Fluctuations in the Stochastic Model

At membrane potentials just below the threshold for initiation of action potentials, stellate cells generate membrane potential fluctuations with a dominant frequency typically in the 5–10 Hz range [14],[16]. Our previous experimental studies using HCN1 knockout mice indicate that, at any given membrane potential, HCN1 channels are not required for fluctuations in this frequency range, but rather HCN1 channels suppress low-frequency components of membrane potential activity [18]. However, the amplitude of the theta frequency fluctuations becomes larger with depolarization towards the spike threshold and if the absolute value of the membrane potential is not accounted for, then deletion of HCN1 channels can appear to reduce the amplitude of membrane potential fluctuations by lowering the most depolarized potential at which fluctuations can be maintained without triggering action potentials [18]. These results contradict proposed deterministic models for the generation of theta frequency fluctuations by stellate cells [17],[26] and also suggest how failure to account for differences in membrane potential could lead to the conclusion that block of HCN channels abolishes theta frequency fluctuations [25]. Nevertheless, it has yet to be shown whether these experimental observations can be accounted for in a theoretical model.

**Figure 2:**
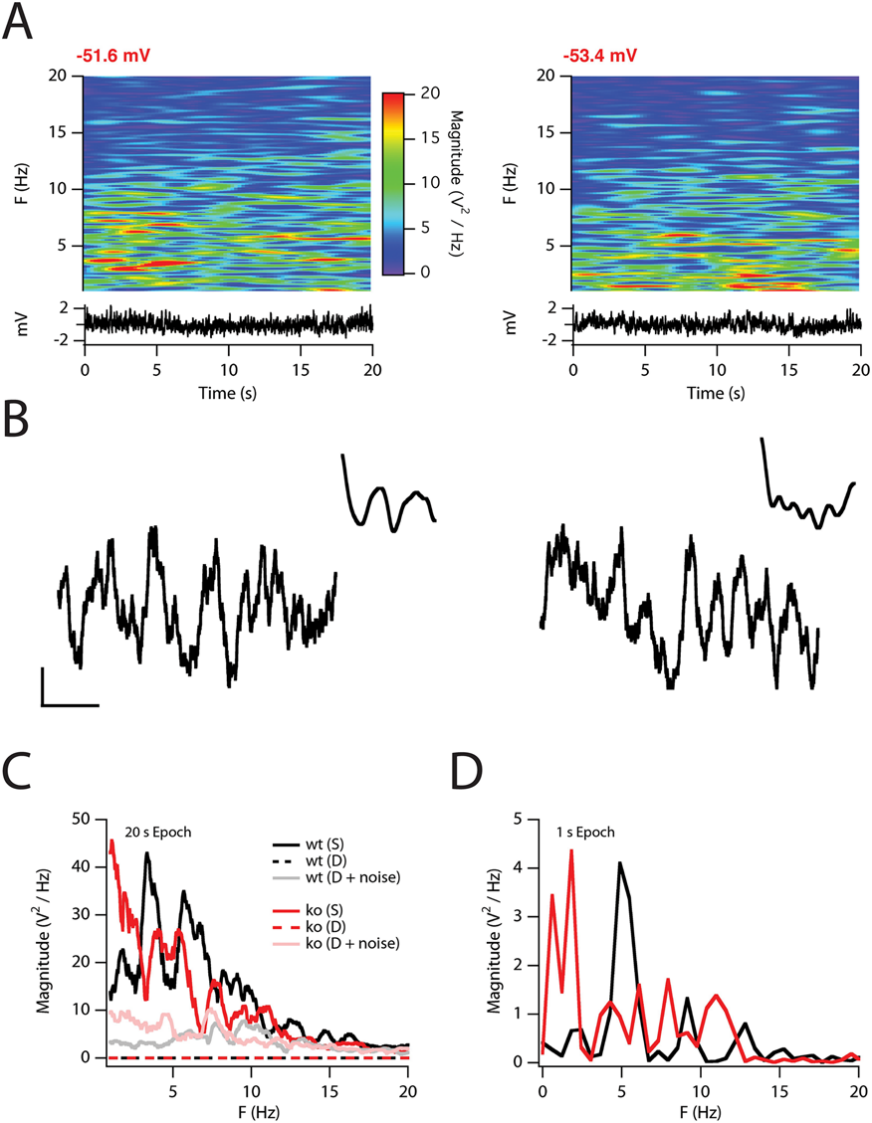
Stochastic gating of ion channels produces perithreshold membrane potential fluctuations. (A) Pseudocolored plots (top) show spectrograms of the membrane potential aligned to the corresponding membrane potential recordings (bottom) of responses to 20 s duration, subthreshold current injection into the wild-type (left) and knockout (right) model. The mean membrane potential is stated in red above the spectrogram. (B) Selected 1 s epochs of membrane potential for the wild-type (left) and knockout (right) model. Insets show the autocorrelation of the membrane potential. Scale bars: 1 mV, 0.2 s. (C) Power spectra for the entire 20 s simulation for the wild-type (black) and knockout (red) models. (D) Power spectra for the membrane potential traces in B, left (black) and B, right (red).

We first examined the membrane potential of the stochastic models during injection of constant current of amplitude adjusted to the maximum possible without triggering action potentials (Figure 2). For the wild-type and knockout versions of the model this corresponded to respective mean membrane potentials of −51.6 and −53.4 mV. At these membrane potentials, the stochastic stellate neuron models show large fluctuations in membrane potential (∼3–4 mV peak to peak; Figure 2), whereas the otherwise identical deterministic models show no fluctuations (Figure 2C). We found that the membrane potential fluctuations recorded over long epochs (20 s) are spectrally complex, but show peak activation between 3–10 Hz consistent with previous observations in stellate neurons *in vitro* [18],[42]. Some previous studies have analyzed brief epochs in which the membrane potential fluctuations appear to be coherent oscillations [16],[25],[43]. Consistent with these studies, we also find that short epochs of membrane potential, recorded from simulations with the stochastic models, reveal clear autocorrelation peaks (Figure 2B) and dominant frequency components in the theta frequency range (Figure 2C and 2D).

Removal of the fast and large component of I_h_ in the knockout model resulted in an apparent shift in the peak of the spectral density to lower frequencies (<5 Hz) similar to previous experimental results in HCN1 knockout mice [18] (Figure 2C and 2D). By contrast, measurements made when controlling for membrane potential between the models, reveal that the knockout model has larger amplitude fluctuations (V_avg_ = −53.7 mV, simulation time = 3 s; σ_WT_ = 0.37 mV σ_KO_ = 0.47 mV; see also Figure S2), also consistent with experimental data [18]. In further agreement with previous experimental data [18], these effects can be explained by the ability of HCN channels to reduce the membrane impedance at low frequencies (Figure S2). As predicted by changes in impedance, responses to a white noise current stimulus, with standard deviation matched to the current noise recorded in the stochastic model, were enhanced in the deterministic knockout model compared with the equivalent wild-type model (Figure 2C). Phase plots of the relationship between membrane current and voltage during perithreshold fluctuations, revealed that I_h_ is a minor contributor (Figures S3) to the net membrane current changes that drive fluctuations. Thus, the stochastic model accounts well for the properties of subthreshold fluctuations and their dependence upon HCN1 channels reported previously [16],[18],[21],[25],[42]. This model is consistent with perithreshold fluctuations arising from interaction of stochastically gating ion channels other than HCN channels (Figure S4) [21], but with the amplitude and spectral properties of the fluctuations shaped by the presence of HCN channels and dependent on the average membrane potential at which the fluctuations are examined.

### I_h_ Determines the Stability of the Perithreshold Membrane Potential

**Figure 3:**
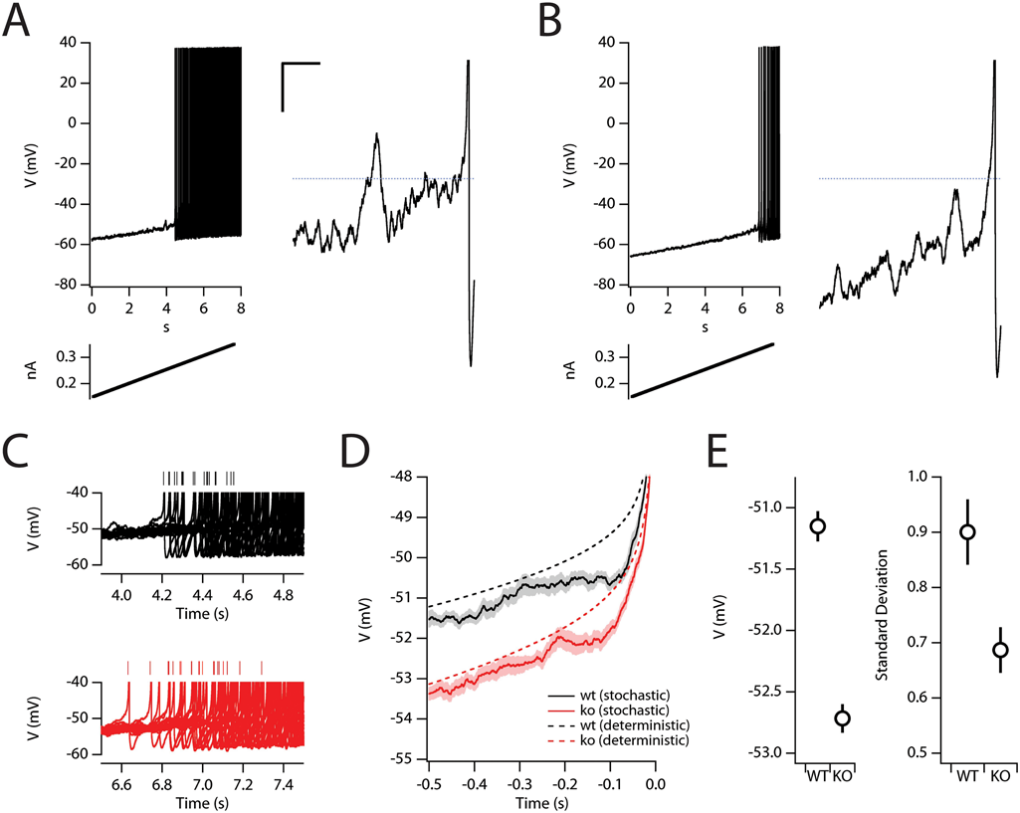
I_h_ enhances perithreshold stability. Example membrane potential responses (top) of the wild-type (A) and HCN1 knockout (B) model to injections of a suprathreshold ramp current (bottom). The region indicated by the box is shown to the right on an expanded scale. Dashed blue line is at −50 mV. Scale bars: 5 mV, 0.1 s. (C) Overlaid membrane potential response to ramp current injection for several trials (n = 20) for the wild-type (black) and knockout (red) models. (D) For each trial the membrane potential was aligned to the time of the first spike. The mean response for the wild-type (black) and HCN1 knock-out models (red) is plotted for both the deterministic (dashed lines) and stochastic models (solid lines). Shaded areas indicate the standard error of the mean. (E) The mean (left) and standard deviation (right) of the spike-triggered membrane potential from −0.5 to −0.1 s prior to the action potential.

The most depolarized average membrane potential that can be maintained without initiation of an action potential appears to determine the maximal observable amplitude of membrane potential fluctuations and is altered both in the HCN1 knockout model (Figure 2) and in experimental recordings of stellate cells from HCN1 knockout mice [18]. To further assess the stability of the membrane potential prior to action potential initiation we injected slow, ramp-like currents that crossed spike threshold for both the wild-type (Figure 3A) and knockout (Figure 3B) versions of the model. We averaged the membrane potential from several sweeps in a time window 0.1–0.5 s before the initial action potential for each trial (Figure 3E). The spike-triggered averages (Figure 3D) revealed that removal of the HCN1-like current from the model causes spikes to initiate from a more hyperpolarized membrane potential (wild-type: −51.15+/−0.12 mV; knockout: −52.72+/−0.12 mV; P = 4×10^−11^; N = 20 total trials; Figure 3E). This difference between the wild-type and knockout models is independent of stochastic channel gating (Figure 3D), but is to be expected from the increased rate of depolarization resulting from the reduced membrane conductance following removal of HCN1 channels. However, for both of the deterministic models the membrane potential follows a more depolarized trajectory than in the corresponding stochastic models (Figure 3D). This is consistent with spontaneous membrane potential fluctuations in the stochastic models triggering action potentials relatively early during the ramp current. Consistent with the difference in responses to DC current injection (Figure 2), during the time-window preceding the spike, the more depolarized potentials in the wild-type model are associated with an increased standard deviation of the membrane potential due to stochastic channel gating (wild-type: 0.90+/−0.06 mV; knockout: 0.69+/−0.04 mV; P = 0.005; Figure 3E). The shift in membrane potential stability was accompanied by a small increase in the standard deviation of the time of the first action potential in the stochastic HCN1 knockout model (wild-type: 0.119±0.008 s; knockout: 0.152±0.015 s; Figure 3C; P<0.05, N = 60 simulations), suggesting that HCN1 channels may increase the reliability of spike timing as well as the stability of the sub-threshold membrane potential.

### Clustered Patterns of Spiking Emerge When Models Contain Stochastically Gating Ion Channels

**Figure 4:**
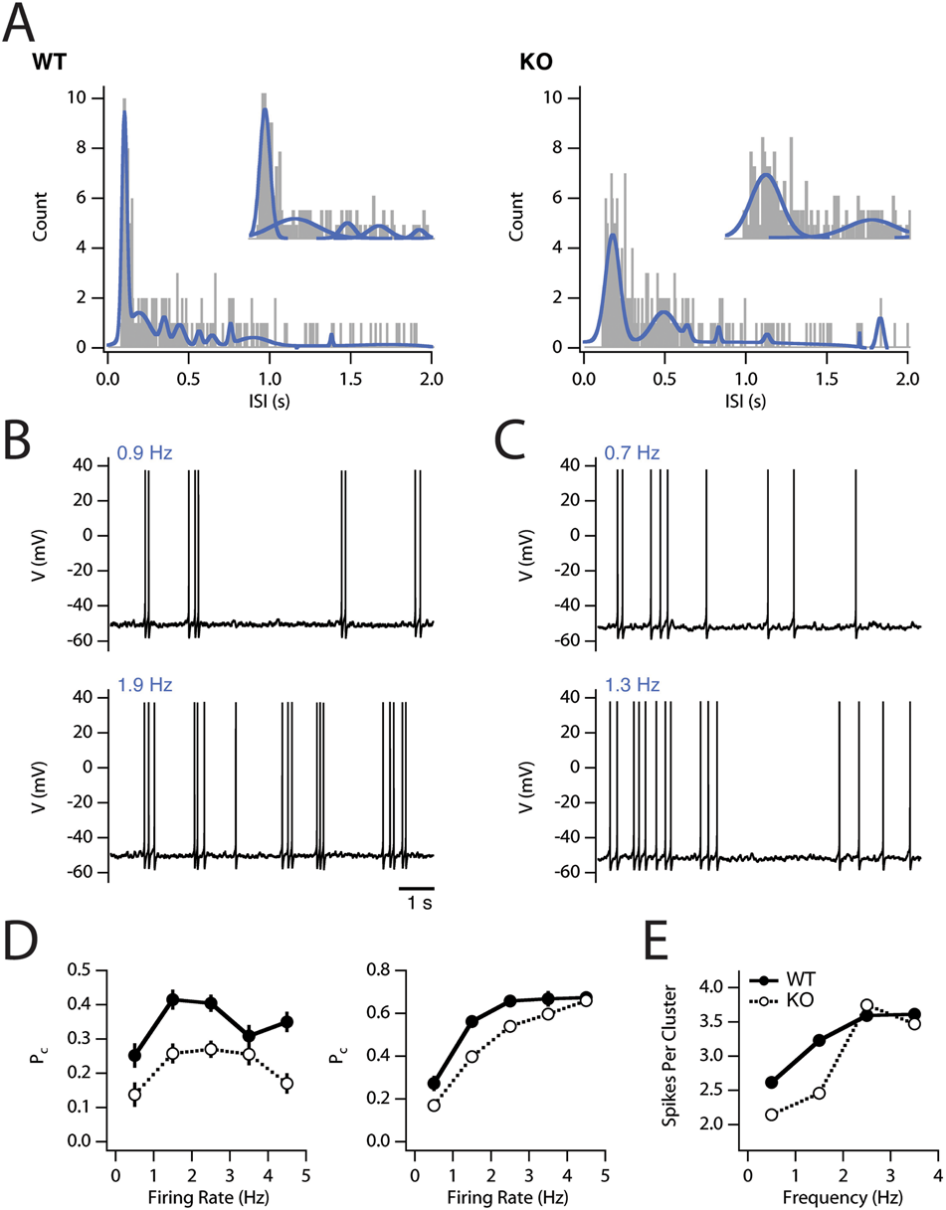
Clustered spiking in the stochastic model. (A) Examples of interspike interval histograms calculated from long duration simulations (150 s) of the response of the wild-type (WT; left) and knock-out (KO; right) models to DC current injection. In both examples the mean firing rate is in the 1–2 Hz range. ISI distributions were fit with multiple Gaussians (solid blue lines). Insets show individual peak fits for the 0–0.6 s interval of the histogram. (B–C) Examples of 10 s duration epochs of membrane potential activity from simulations with the wild-type (B) and knockout (C) models. Average firing rate for the trial is stated in blue. (D) P_c_ is plotted as a function of average firing rate for the wild-type (closed symbols) and knockout (open symbols) models using the ‘stringent’ clustering definition (left panel) and the ‘relaxed’ clustering definition (right panel). Several hundred, 16 s duration simulations of the partially stochastic model (Figure S6) were used to provide detailed sampling. (E) Number of spikes per cluster is plotted for a subset of the data.

When stellate cells experience maintained depolarizing currents that drive action potential firing at mean frequencies less than 5 Hz, the pattern of firing is characterized by clusters of action potentials at a relatively high frequency (8–14 Hz) interspersed with silent periods [14],[18],[24]. We determined the conditions for initiation of spikes with mean frequencies less than 5 Hz, at which clustered spike patterns might be expected. In the deterministic model the transition from silence to continuous action potential firing occurs when the amplitude of the injected current is increased above 258.4 pA and 320.5 pA for wild-type and knockout configurations, respectively. For the deterministic models this transition corresponds to a sharp transition from silence to repetitive spiking at ∼6 Hz (wild type) and ∼3 Hz (HCN1 knockout) and clustered spike patterns were not observed (Figure S5). By contrast, the current threshold for the transition between silent and spiking states was ∼246 pA and ∼308 pA for the stochastic versions of the wild-type and HCN1 knockout models, respectively. In both stochastic models, arbitrarily low firing frequencies could be obtained when the injected current was just above this threshold. When the mean frequency of action potentials was less than approximately 5 Hz, then both stochastic models generated clustered patterns of spikes (Figure 4). Thus, stochastic ion channel gating enables clustered patterns of spikes to emerge during firing at low frequencies in response to input currents that are of insufficient amplitude to initiate action potentials in the corresponding deterministic model.

We next examined in detail the patterns of spiking that emerge when constant current injected into the stochastic model drives low-frequency action potential firing (Figure 4). Consistent with electrophysiological results [18],[24],[44], we find that the interspike interval (ISI) distribution of the stochastic model in response to constant current injection is multimodal, being characterized by both a dominant, short ISI mode as well as a wide distribution of long ISIs (Figure 4A). However, in the knock-out model this short latency peak is much broader than in the wild-type model (Figure 4A). Closer examination of the model behavior across a range of average firing frequencies revealed the characteristic tendency of stellate neurons to fire clustered action potentials (Figure 4B and 4D). The knockout version of the model reveals a lesser tendency to fire spikes in clusters (Figure 4C and 4D), consistent with the broadening of the short latency peak in the ISI histogram (Figure 4A). We quantified the probability of clustering (P_c_) with definitions used previously for experimental data (see Materials and Methods; [18]). For the wild-type stellate neuron model P_c_ depends upon average firing frequency and peaks at intermediate (1–3 Hz) frequencies (Figure 4D). Importantly, P_c_ is significantly reduced in the knockout model at intermediate average firing rates (Figure 4D). Finally, as in experimental recordings, the average number of spikes per cluster in the stochastic models is quite variable and depends on the average firing frequency (Figure 4E).

### HCN Channels Influence the AHP Waveform in Stochastic and Deterministic Models

**Figure 5:**
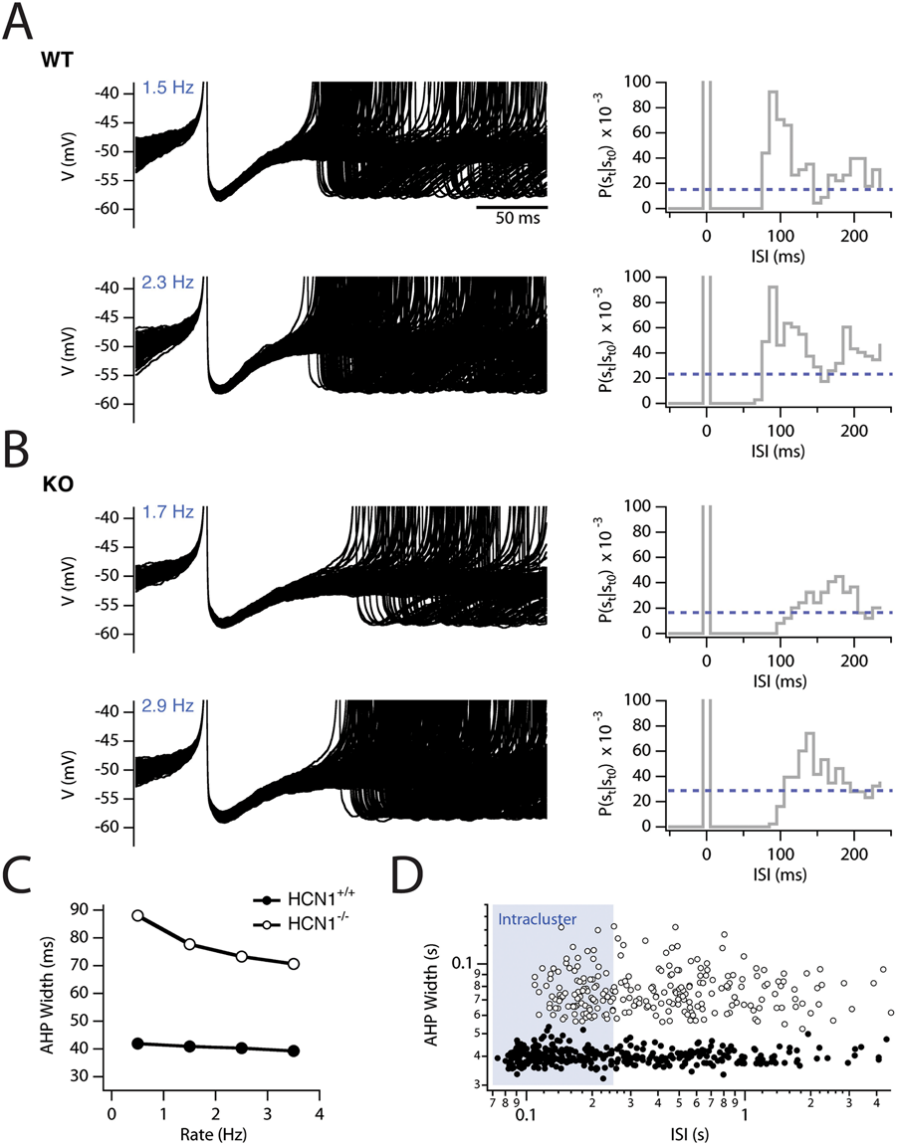
Recovery from the AHP is influenced by I_h_ and reflects spike clustering. (A–B) Overlaid action potentials (left) and corresponding conditional spike probabilities (right, truncated at P = 0.1) from long simulations (150 s) in which constant current was injected to the wild-type (A) and knockout (B) models. Average firing rate from the selected trials is indicated in blue. (C) Average width of the AHP at −52 mV for the wild-type (closed circles) and knockout (open circles) spikes. (D) For a representative trial (upper panels in A and B) a log-log plot of the AHP width against the succeeding ISI for wild-type (closed circles) and HCN1 knockout (open circles) versions of the model.

We previously demonstrated that I_h_ accelerates the repolarization from the AHP in stellate neurons, while overall shorter AHPs predict an increased tendency of neurons to fire clustered patterns of action potentials [18]. Similarly, the half-width of the AHP in the wild-type stochastic model was independent of the average frequency of spike firing (Figure 5C). In contrast, after the simulated removal of HCN1 channels, the AHP half-duration was broader and varied as a function of average spike frequency (Figure 5C), just as in experimental recordings from stellate neurons in HCN1 knockout mice [18]. The increase in duration of the AHP following removal of HCN1 channels was found in both stochastic and deterministic (Figure S5) versions of the model indicating that this role of I_h_ does not require stochastic gating of the membrane ion channels. To quantify spike initiation following the AHP we calculated the conditional probability that a spike occurred at a time *t* following a previous spike at time *t0* (P(s_t_|s_t0_)) [45]. For spike trains generated by the knockout model (Figure 5B, right panels), the latency to the increase in P(s_t_|s_t0_) following a spike was increased and the magnitude of the change in P(s_t_|s_t0_) was reduced from more than 6 fold to less than 3 fold compared with spike trains generated by the wild-type model (Figure 5A, right panel). These changes are correlated with the reduction in P_c_ observed in simulations of the knockout model across a range of firing frequencies (Figure 4D).

Together, these simulations indicate that deterministic or stochastic versions of our model stellate neuron are sufficient to account for the resting membrane properties, subthreshold stability of the membrane potential and the sensitivity of these properties to alteration of I_h_. However, only the version of our model containing stochastically gating ion channels is able to further account for the spontaneous emergence of membrane potential fluctuations at potentials near threshold. Moreover, the stochastic models produce clustered patterns of action potentials similar to spike patterns recorded from stellate neurons from wild-type and HCN1 knockout mice. Since our characterization of the stochastic models suggest that they provide a remarkably good account of experimental observations of both the resting and active properties of entorhinal stellate neurons, we went on to use these models to investigate how stochastic ion channel gating influences spike initiation and the generation of distinctive clustered patterns of action potentials.

### Clustered Firing Patterns Involve Brief Action Potential Dependent Changes in Firing Probability

How do the clustered patterns of action potentials emerge and why do they require stochastic ion channel gating? In a deterministic neuron, clusters or bursts of action potentials arise through modulation of spiking by slow changes in the state of one or more ion channels [46],[47]. Indeed, such a deterministic mechanism has previously been proposed to account for clustered patterns of action potentials fired by entorhinal stellate neurons [26]. By this account, stochastic ion channel gating may lower the threshold for spike generation, but is not essential for the generation of clustered patterns of activity. However, stochastic ion channel gating may permit mechanisms for control of spike patterns that are not possible in deterministic models. In particular, whereas initiation of an action potential in a deterministic neuron is binary, with a clearly defined threshold, for stochastic neurons fluctuations in ion channel activity can lead to cancellation of a spike even when the deterministic threshold is crossed. At the other extreme spikes can be initiated in conditions that are well below the deterministic spike threshold [8],[9]. Therefore, in a stochastic neuron there is no clearly defined boundary between a spiking and a non-spiking state and thus spike initiation should be considered probablistic rather than binary.

**Figure 6:**
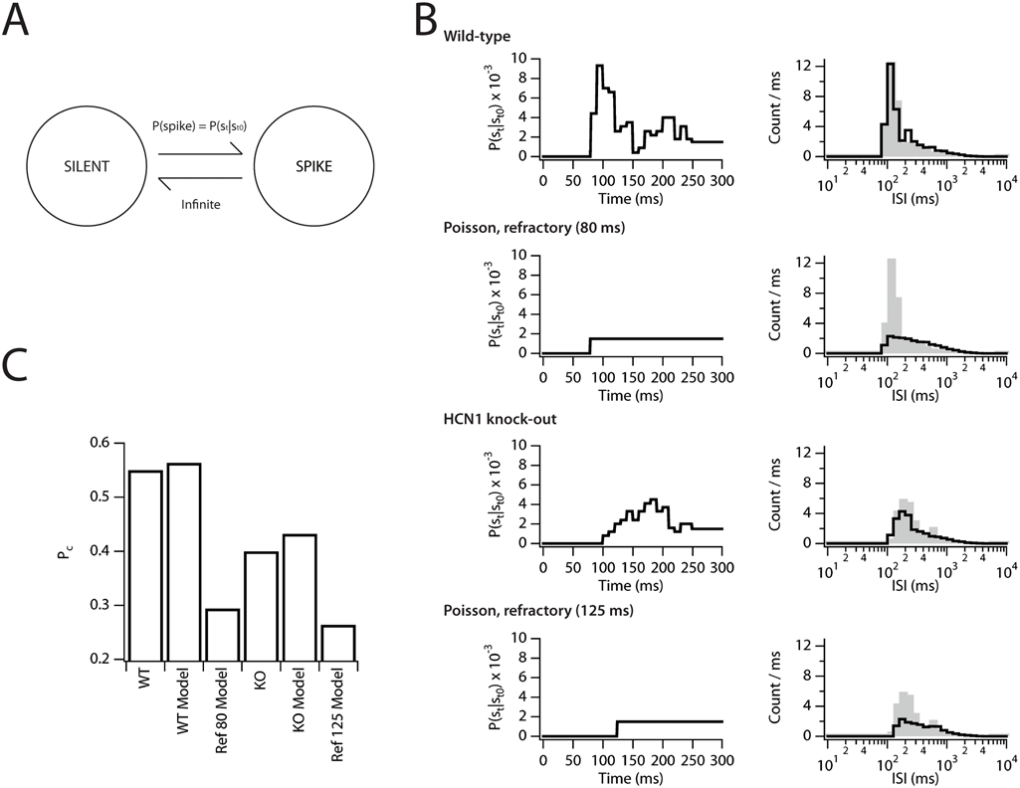
A transient increase in firing probability during recovery from the AHP is sufficient to account for clustered spiking. Spiking was modeled as a stochastic point process. (A) According to the point process model the stellate neuron makes stochastic transitions between a silent and spiking state with a probability determined by P(s_t_|s_t0_) and an instantaneous transition from spiking back to silence (see text for explanation). (B) We considered 4 possible P(s_t_|s_t0_) functions (left panels). The “Wild-type” and “Knockout” P(s_t_|s_t0_) curves are taken directly from data obtained with the corresponding stochastic models. In addition, Poisson processes with refractory periods of 80 and 125 ms were considered. ISI histograms were generated from long simulations (1000 s) (right panels). For comparison an ISI distribution from the wild-type stochastic stellate model is also plotted (gray bars). (C) For each of the spike trains simulated with the stochastic point-process model (“WT model” and “KO model”) P_c_ was calculated and plotted along with the P_c_ values in the 1–2 Hz bin of simulations in the wild-type (“WT”) and knockout (“KO”) versions of the stochastic stellate model.

The probabilistic nature of spiking in the stochastic model leads to a simple alternative mechanism for generation of clustered patterns of spikes, whereby the transient elevation in the probability of spiking following a previous action potential is sufficient to produce patterned output (Figure 6). According to this mechanism, changes in the recovery from a spike would alter the pattern of spikes by modifying the spike probability immediately following the refractory period (Figure 6B). As a result, the activation of ion channels during each action potential and its associated AHP can be independent of the position of the action potential within or outside a cluster. Several lines of evidence support this probabilistic mechanism.

First, conditional probability distributions, P(s_t_|s_t0_) (Figure 5A and 5B, right panels; also see Materials and Methods), reveal that the wild-type version of the model produces clustered action potentials by elevating the conditional probability of firing a spike, P(s_t_|s_t0_), over the steady-state probability, P(s_t_), for a brief period of ∼50 ms following a spike (Figure 5A, right panels). Moreover, the reduction in P_c_ in the HCN1 knockout model is correlated with a decrease in P(s_t_|s_t0_) (Figure 5B, right panels) as required by a probabilistic mechanism for clustered firing (Figure 6B).

Second, the number of spikes within a cluster is variable for a particular firing rate (*e.g.* 3.11±1.7 spikes per cluster for 1.6 Hz) and depends upon the average firing rate in both our model (Figure 4E) and experimental data [18]. This suggests that the number of spikes in a cluster is probabilistic and is consistent with a stochastic model of spike generation, but distinct from previous deterministic models [26].

Third, in a deterministic mechanism the half-width of the AHP should systematically vary with position in the cluster and should determine the succeeding ISI when terminating a cluster. Thus, on a spike-by-spike basis we would expect the AHP to correlate with the subsequent ISI. However, we find no such correlation in spike trains from either the wild-type or knockout models (Figure 5D). Nonetheless, in both population data from experiments and in different versions of the stochastic model the AHP half-width correlates with P_c_. These observations therefore support our conceptual model of spike patterning and suggest that there may be a common ionic basis that regulates the time course of both the AHP and P(s_t_|s_t0_).

**Figure 7:**
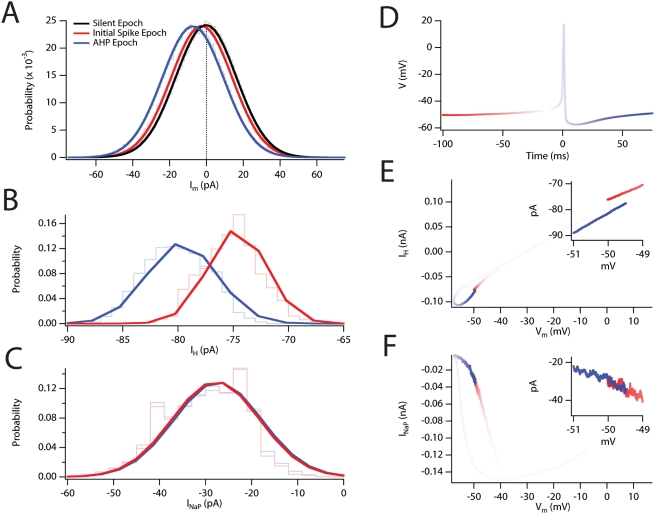
I_h_ during AHP recovery enhances spike probability and clustering. (A) Probability density plots for the magnitude of the net ionic current within the voltage range −49.5 to −50.5 mV taken from epochs in which no action potentials occurred (“Silent”; black), preceding the initial spike of a cluster (“Initial Spike”; red), or, during recovery from the spike AHP (“AHP”; blue). Each plot is fit with a Gaussian function, which was used to estimate the standard deviation of the distribution. Areas were normalized to P = 1 and all distributions had nearly identical properties (σ_steady-state_ = 16.6 pA, σ_AHP_ = 16.6 pA, σ_silence_ = 16.5 pA). (B–C) Probability density plot for I_h_ (B) and for I_NaP_ (C) during the same simulation epoch as in A. (D) Color-coded plot of the average membrane potential for all action potentials. Transition from red to blue color applies to E and F. Solid lines are derived from fits of Gaussian functions. (E) Phase plot of the mean I_h_ during the spike. (F) Phase plot of the mean I_NaP_ during the spike. (E–F) Insets focus in on the region of membrane potential selected for the plots in A–C.

Fourth, to generate activity patterns that take place over relatively long time scales, such as spike clusters, a deterministic model requires relatively slow changes in the state of the model and at least one of the model parameters must vary as a function of a spike's location within a cluster. By contrast, the probabilistic mechanism of spike clustering does not require slow changes in model parameters beyond the recovery period from the AHP (Figure 6). Consistent with this prediction we find that the distribution of currents during AHP recovery is not different between the first spike in a cluster and all other spikes regardless of their position (see below, Figures 7 and S8).

Fifth, the conditional spike probabilities (P(s_t_|s_t0_)) are sufficient to generate spike trains with interspike interval histograms and clustered patterns of spikes that are indistinguishable from spike trains generated by the biophysical neuronal models (Figure 6B and 6C). Thus, P(s_t_|s_t0_) can fully characterize the spike train. By contrast, if there were higher-order correlations in the spike probabilities, as would be the case in any deterministic model of clustered spiking, then the conditional probabilities would differ for each spike and no single set of conditional spike probabilities would fully characterize the spike train [45].

### Transient Increases in Spike Probability Following AHPs Are Associated with an Inward Shift in the Balance of Membrane Currents

In principle, the transient increase in the probability of action potential firing that occurs following recovery from the AHP could arise though a number of mechanisms: (1) A transient shift in the balance of membrane currents that together determine the overall direction and rate of change of the membrane potential; (2) A change in the stochastic current fluctuations that act as the noise source that enables probabilistic spike firing; or (3) A reduction in the threshold for spike initiation.

To address the first possibility, we evaluated the membrane current at a narrow range of membrane potentials (−50.5 to −49.5 mV), just below the voltage threshold for spike initiation (Figure S8). In the stochastic model the membrane potential enters this range during silent epochs when spikes are not initiated, immediately before initiation of the first spike in a cluster and in the epoch following recovery from the AHP when a subsequent spike may or may not be triggered. We therefore assigned each membrane potential measurement to one of three different classes (Figure 7A): a 50 ms windows prior to spike initiation from steady state (red); during AHP recovery of all spikes without regard to their position within a cluster (blue); and silent epochs during which no spiking occurred during or in the subsequent 100 ms (black). For each point within these time windows we sampled the membrane current if the membrane voltage was within the range −50.5 mV to −49.5 mV and then generated histograms of the membrane current for each epoch. Comparison of these three cases revealed that spike initiation from steady state is associated with a small but significant inward shift in the net ionic current relative to periods of silence (Figure 7A). The small shift in the mean current is consistent with the low average firing frequency at steady state (*i.e.* low P(s_t_)). By contrast, the recovery from the AHP is associated with a larger shift (∼8 pA) of the net membrane current in the inward direction (Figure 7A), consistent with the increase in P(s_t_|s_t0_) relative to P(s_t_) following AHP recovery and with the shift observed for spikes that initiate clusters (Figure S8). Thus, during the period following recovery of the AHP, the membrane experiences on balance a greater net inward current at potentials approaching threshold, driving further depolarization of the membrane potential and spiking.

We also evaluated whether other mechanisms might contribute to the change in firing probability following recovery from the AHP. Importantly, we found no difference in the standard deviation (σ) of the membrane current prior to initiation of spikes from steady state (σ = 16.6 pA), compared with AHP recovery (σ = 16.6 pA) or silence (σ = 16.5 pA), indicating that stochastic current fluctuations have a similar magnitude in each condition (Figure 7A). Moreover, there was no correlation between the membrane potential at which we detected spike initiation (see Materials and Methods) and the preceding ISI for either the wild-type or knockout models (R<1×10^−4^; Figure S7), indicating that the brief elevation in P(s_t_|s_t0_) is not due to an alteration in the voltage threshold following a previous spike. Thus, the shift in average membrane current, as opposed to a change in the stochastic current fluctuations or spike threshold, appears to be the major determinant of increased firing probability following the AHP.

### Slow Activation and Deactivation of I_h_ Determines the Time Window for Increased Spike Probability Following an AHP

Since our experimental and modeling data indicates that HCN channels influence both the AHP and clustered spiking, we asked whether changes in I_h_ during the AHP could account for the shift in membrane current that underlies the increase in P(s_t_|s_t0_) relative to P(s_t_). Importantly, the shift can be fully explained by an increase in the amplitude of I_h_ during AHP recovery (Figure 7B). By comparison another current important for spike initiation, the persistent sodium current (I_NaP_), shows no change (Figure 7C). Consistent with this explanation, phase plots for I_h_ (Figure 7E) and I_NaP_ (Figure 7F) during an action potential, reveal an increased I_h_ density associated with recovery from the AHP.

**Figure 8:**
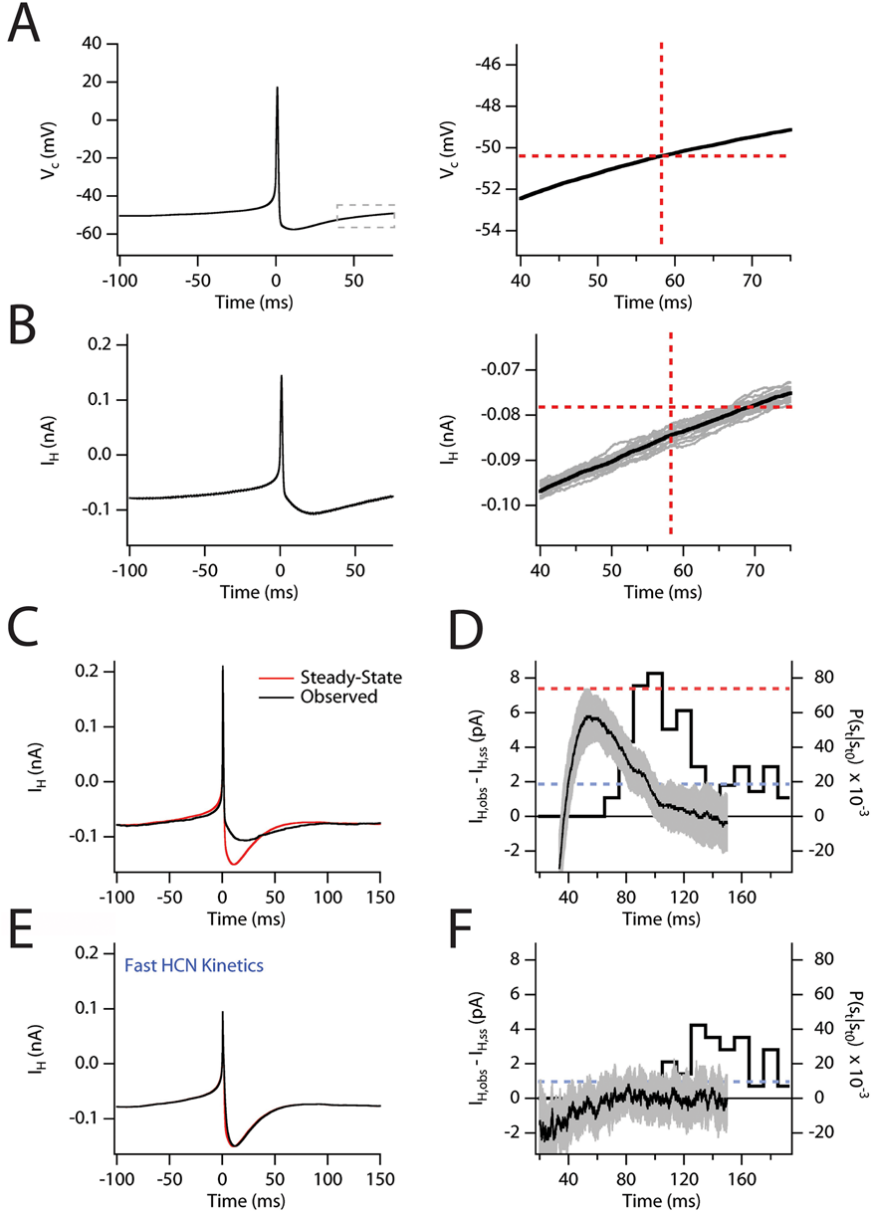
Elevated I_h_ during AHP recovery correlates with increased spike probability. (A) The voltage command (V_c_) waveform used for voltage-clamp simulations (left). The voltage command in the region indicated by the box is also shown on an expanded scale (right). Horizontal line indicates initial value of the command potential. Vertical line indicates time at which command returns to its initial value. (B) Isolated I_h_ during voltage-clamp of the model to the command potential in A (average of 10 simulations). (B, right) Isolated I_h_ during voltage command return to steady state. The plot corresponds to the region of the voltage command highlighted in the right hand panel of A. Solid black line indicates average of 10 simulations shown individually in gray. Vertical and horizontal lines as in A. (C) Observed I_h_ (red) is plotted along with the steady-state I_h_ density expected at each potential in the command waveform. (D) Plot of the difference between the observed and expected steady-state I_h_ (I_obs_−I_ss_) during the period of AHP recovery in the command potential. Superimposed is the plot of the probability of an action potential, P(s_t_|s_t0_), for the clustering simulations in Figure 5. Dashed blue line indicates the average spike probability during the simulation. Red dashed line indicates the expected spiking probability when the injected current is increased by 7 pA. Shaded grey area indicates standard error of the mean of I_obs_−I_ss_. (E) The observed I_h_ (red) after increasing the microscopic gating rates is plotted along with the steady-state I_h_ density expected at each potential of the command waveform. (F) Plot of I_obs_−I_ss_ during the period of AHP recovery in the command potential for the case for the fast I_h_ shown in E. Superimposed is the plot of the probability of an action potential (P(s_t_|s_t0_)) for a clustering simulation (150 s DC stimulus) with the same injected current as the data in D.

Are the kinetics of I_h_ important for the relatively brief increase in P(s_t_|s_t0_) that appears to underlie generation of clustered patterns of activity (Figures 5 and 6)? Simulated voltage-clamp of isolated I_h_ using a command potential based upon the action potential waveform (Figure 8), revealed an increased density of I_h_ following recovery from the AHP (Figure 8B). Comparison of the observed I_h_ (I_obs_) with the current density predicted from the steady-state I–V relationship for I_h_ (I_ss_), revealed that while I_obs_ was less than I_ss_ at time points corresponding with the peak of the AHP, during the return phase of the AHP I_obs_ is larger than I_ss_ (Figure 8C amd 8D). This transient elevation in I_h_ relative to steady-state precedes the time course of P(s_t_|s_t0_) with an expected lag for action potential initiation and detection (Figure 8D). To determine if this shift in net membrane current could cause the shift in firing probability, we simulated an increase in the injected current by the peak value of I_obs_−I_ss_. The increase in P(s_t_) (dashed red line; Figure 8D) during this simulation relative to P(s_t_) under the control simulation (dashed blue line; Figure 8D) accurately predicts the peak of P(s_t_|s_t0_). Thus, a brief change in the net inward current due to I_h_ during the AHP appears to be sufficient to explain the magnitude and time course of P(s_t_|s_t0_).

### The Slow Gating Kinetics of I_h_ Are Important for Clustered Spiking in the Model

To directly test the influence of the slow gating kinetics of I_h_ on action potential clustering we scaled the forward and reverse rates of the closed-open transition of I_h_ (Figure S9). While scaling the kinetics did not alter the magnitude of the steady-state current, it did allow I_h_ to equilibrate to the membrane potential during recovery from the AHP (Figures 8E and S9) and significantly reduced the short-latency (∼100 ms) peak in P(s_t_|s_t0_) (Figures 8F and S9). This reduction in spike probability following a prior spike resulted in a 33% reduction in P_c_ for a 1–2 Hz average firing rate. However, changing the kinetics of I_h_ complicates this analysis and likely leads to an underestimate of the effect. For example, the change in kinetics leads to a 10% reduction in the AHP half-width and increases the stochastic fluctuations in I_h_ about its mean, both of which effects could increase P_c_. Stochastic gating of HCN currents is not necessary for clustered spiking (Figure S6). Thus, we also ran simulations with fast, deterministic HCN channels to prevent the increase in fluctuations and found that P_c_ was reduced 40% to 0.33, close to the theoretical minimum of 0.29 for a refractory Poisson process where P(s_t_|s_t0_) is equal to P(s_t_) (Figure 6).

Together, these data suggest that activation of I_h_ during the AHP is an important determinant of both the AHP half-width and the clustering of action potentials. Given the relatively slow kinetics of I_h_ the closing of HCN channels lags the depolarization of the membrane on the tail of the AHP and I_h_ fails to equilibrate to the membrane potential. As a result, the AHP recovery is associated with a transient increase in I_h_ relative to steady-state that contributes to an increase in the probability of action potential initiation. Moreover, this effect is robust across a range of channel kinetics tested (Figure S9). However, due to their relatively small single channel conductance [48], changes in mean HCN current act primarily as a DC bias current, rather than as a noise source.

### The Stochastic Model Can Account for Firing Properties of MEC Neurons *In Vivo*

**Figure 9:**
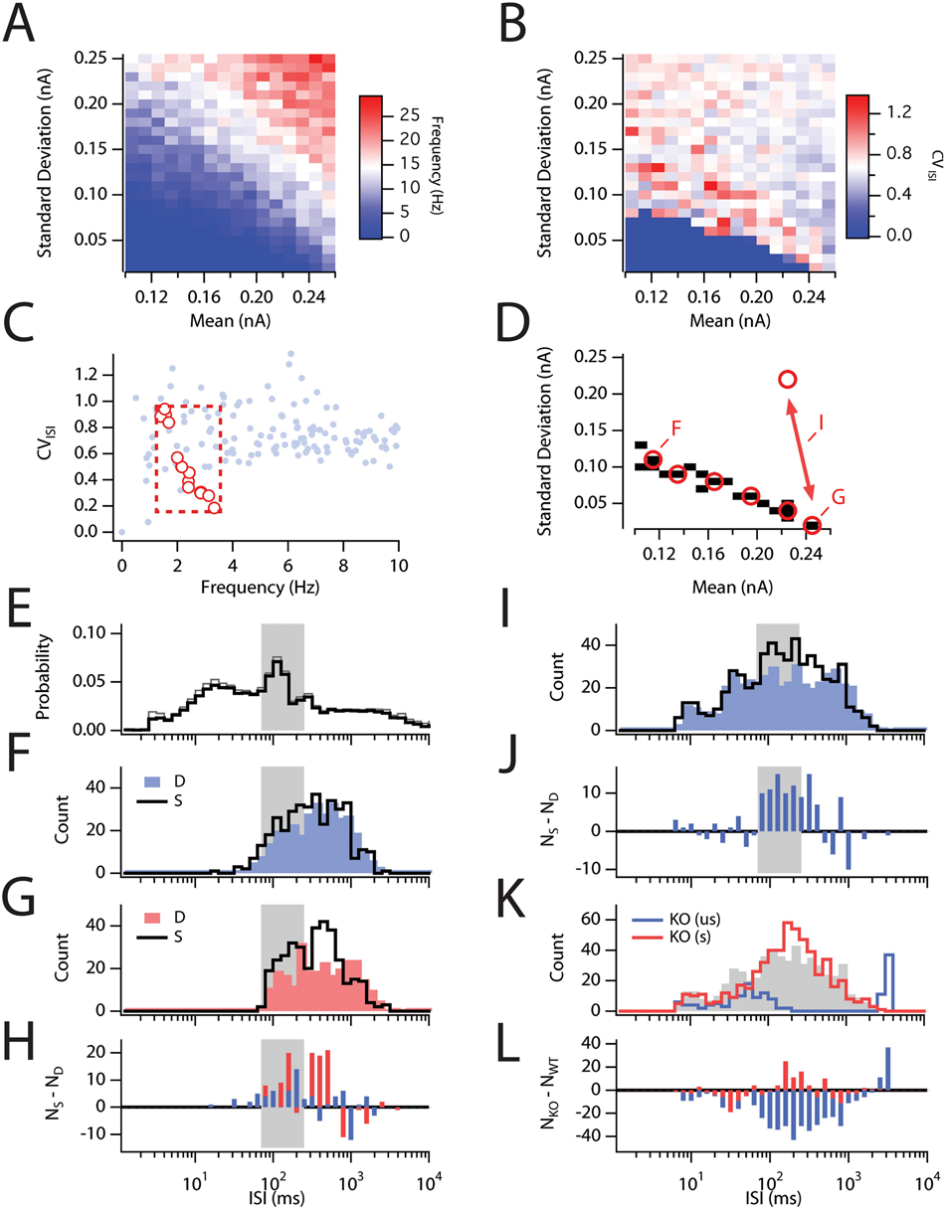
Effects of stochastic channel gating alter the response to stellate cells to naturalistic stimuli. (A,B) Plot of mean spike frequency (A) and coefficient of variation of the ISI distribution (B) as a function of the mean and standard deviation of band limited white noise inputs obtained from 5 s duration simulations (N = 384). (C) CV plotted as a function of mean firing frequency for the same data shown in A and B. The frequency and CV of several recordings (see [49]) from neurons in the superifical layers of the medial entorhinal cortex *in vivo* are plotted for comparison (red dots). These values from *in vivo* data were used to define a region of stimulus space selected for further analysis (red box). (D) A masked plot of stimulus space shows the simulations that resulted in values within the red box defined in C. Longer simulations (150 s) were run for the points indicated in red using both the deterministic and stochastic models. (E) The mean ISI probability density for experimental recordings plotted in C. Gray shaded region indictates the range of ISIs for spike clusters (see text). (F, G) ISI histograms obtained from simulations with the deterministic (“D”) and stochastic (“S”) versions of the model using input statistics at the extrema of the plot in D (indicated by “F” and “G”). (H) The difference in spike counts between the D and S simulations for the data plotted in F (blue) and G (red). The stochastic model shows a selective redistribution in the probability of spiking that produces an increase in the clustering interval (shaded region) and a decrease at longer ISI intervals. (I) ISI histograms obtained from simulations with deterministic (blue) and stochastic (black) versions of the model using input statistics that fluctuate randomly between the two states indicated by the double-headed arrow in D. (J) The difference in spike counts between the D and S simulations for the data plotted in I. (K) ISI histogram for response of the knockout model to the unscaled (“us”; blue) and the scaled (“s”; red) poisson stimuli (see text). Gray shaded region is the data from I replotted. (L) The difference in count between the “us” and “s” simulations for the data plotted in K. All histograms use exponentially spaced bins.

Could the stochastic model that we outline here also explain aspects of the firing patterns of neurons in behaving animals? Consistent with this possibility, spike times obtained from *in vivo* single unit recordings [49] show elevations (made clear by exponential bin spacing [50]) in their ISI distribution at around 100 ms (Figure 9E). This ISI resembles the peak of P(s_t_|s_t0_) in simulations of our stochastic model, but unlike the responses of our model to constant current input, the *in vivo* spike trains contain a much broader overall distribution of ISIs. To provide a more realistic comparison between the model and *in vivo* data, we therefore carried out stimulations of the response of the model neuron to simulated synaptic drive.

To reduce the uncertainty of comparing the model output with *in vivo* recordings during which the physiologically relevant inputs are unknown, we first examined a wide region of stimulus space by varying the standard deviation and offset of a band-limited, white noise stimulus (F_max_ = 50 Hz). In this way, we obtained a description of the relationship between properties of the simulated input to the model and the mean frequencies (Figure 9A) and coefficient of variation (CV; Figure 9B) of the ISI distributions generated by the spike outputs from the model. Based on comparison of these data with the frequency and CV of spike trains recorded *in vivo* (Figure 9C), we selected for use in further simulations parameters that generated spike trains with CV and ISI spanning the space covered by the *in vivo* spike data (Figure 9D). For inputs with a large standard deviation and a small offset there were only small differences between output responses of the stochastic and deterministic versions of the wild-type model (Figure 9F; χ^2^-test, P = 0.01). In contrast, for inputs with a large offset and small standard deviation, striking differences were apparent between the responses of the stochastic and deterministic models (Figure 9G; χ^2^-test, P<0.0001). In both cases the stochastic model tends to redistribute the average ISI distribution such that it is enriched for 100–200 ms ISIs, but this effect is greater for the responses to weakly varying inputs (Figure 9H).

Unlike the *in vivo* experimental data, the simulations above did not generate high frequency (>25 Hz) bursts of spikes. However, examination of the stimulus space indicated that high variance stimuli with substantial DC offsets could produce spikes at high frequency (Figure 9A). Since recordings of the local field potential in the medial temporal lobe *in vivo* indicate that the network is characterized by long periods of relatively uncorrelated activity interspersed with brief epochs of highly correlated activity [51], we attempted to mimic these stimulus statistics by assuming that the stimulus can be characterized by a relatively low average variance (characteristic of uncorrelated presynaptic activity) interspersed at random (Poisson) delays (λ = 1 s) with random duration (λ = 200 ms) epochs of high average variance (characteristic of correlated presynaptic activity). This pattern of stimulation is illustrated graphically as a transition between two points in stimulus space (Figure 9D) and resulted in a much broader ISI distribution that more closely matched the *in vivo* data (Figure 9I). Under these stimulus conditions, simulations of the stochastic model also resulted in an ISI histogram enriched for intervals around 100 ms consistent with clustered spiking (Figure 9J; χ^2^-test, P<0.0001).

Finally, we sought to determine predictions the model could make for the *in vivo* distribution of ISIs for MEC stellate neurons in HCN1 knockout mice. Assuming the stimulus conditions reflect properties of the inputs to the MEC *in vivo*, the stochastic model predicts that stellate neurons from HCN1 knockout mice should show reduced average firing rates (F_WT_ = 3.77 Hz; F_KO_ = 1.01 Hz) and less clustered firing (Figure 9K and 9L), but an increase in the fraction of spikes emitted in high frequency bursts (Figure 9K). While we have not found evidence for compensatory changes following HCN1 deletion [18],[27],[28],[38], we nevertheless also considered the possibility that differences in excitability between wild-type and HCN1 knockout mice may be compensated for by homeostatic changes in the average strength of synaptic inputs *in vivo* [52]. Thus, compensating for the shift in the current threshold for spike firing following HCN1 deletion by altering the average offset amplitude of the simulated *in vivo* synaptic input, the stochastic model predicts that MEC stellate neurons from HCN1 knockout mice should show a slight shift in the peak of their ISI distribution of approximately +100 ms (Figure 9K and 9L). In addition, over a range of compensation values all of our simulations (data not shown) suggest that the peak of the ISI distribution in the range of clustered spiking should actually increase in the knockout mice presumably due to the increased impedance of the membrane near threshold (Figures 2, 3, and S2).

## Discussion

Stellate neurons from layer II of the MEC have distinctive membrane properties that are proposed to be central to their function of integrating cortical inputs to the hippocampal dentate gyrus [14]. We find that a biophysical model neuron in which the ionic currents are represented as a population of discrete, stochastically gating individual ion channels provides a unified biophysical account of intrinsic oscillations of membrane potential and clustered patterns of action potential firing recorded from entorhinal stellate neurons. Whereas passive properties at hyperpolarized potentials can be explained when ionic conductances are assumed to be deterministic (Figure 1), the dynamic properties of the membrane potential near the threshold for spike initiation require that ionic conductances be modeled as populations of individual ion channel proteins subject to random fluctuations in conformation (Figures 2 and 3). Patterned action potential firing arises spontaneously in the stochastic model, whereas it is absent from the deterministic model (Figure 4). Clustered spike patterns result from the modification of the probability of action potential initiation for a brief epoch following a spike (Figure 5). The model of stellate cells that we developed implements an example of a general mechanism for controlling patterns of action potential firing by activity dependent changes in spike probability (Figure 6) that can be mediated by HCN channels (Figures 7 and 8). Further simulations with this model suggest conditions in which this mechanism could account for patterns of action potentials recorded from stellate neurons in behaving animals (Figure 9).

### The Patterns of Spike Output from Neurons with Stochastically Gating Ion Channels Can Be Controlled by Activity Dependent Changes in Spike Probability

The rules that determine transformation of synaptic input into patterns of spike output are fundamental to computations carried out within the central nervous system. While models of many cortical neurons take advantage of simplifying assumptions that characterize spike output as an invariant function of synaptic input (*e.g.* [53],[54]), experimental recordings suggest that stellate neurons from layer II of the MEC generate clustered patterns of spike output through intrinsic mechanisms that may not be reducible in this way. In the biophysical model of a stellate neuron that we develop here, a brief increase in spike probability immediately following recovery from a preceding action potential can substantially modify the pattern of spike output. In the low firing frequency regime, spikes can be initiated by random fluctuations of the net membrane current. As a result of the balance of currents near threshold, the low effective membrane conductance, and the relatively large currents that can be produced by individual ion channels, small bias currents can substantially alter the probability of firing by shifting the mean of the net membrane current. This model is sufficient to explain the clustered patterns of spikes that are recorded from stellate cells during injection of constant current (Figure 6). This mechanistic account also provides some suggestion that the tendency of neurons in layer II of the MEC in behaving animals to fire spikes at 5–10 Hz may result from the transient, spike-dependent increase in spike probability that can influence spiking even in the presence of a continuously varying barrage of synaptic inputs (Figure 9).

The model that we develop here differs from a number of other models proposed to explain the integrative properties of stellate neurons. Two previous, biophysically-detailed deterministic models have proposed that cyclic interactions between I_NaP_ and I_h_ are necessary and sufficient to produce perithreshold oscillations [25],[26]. However, this conclusion is not supported by experimental observations from stellate neurons following genetic deletion of HCN1 [18], or pharmacological block of I_h_ [22]. One of these previous biophysical models also produces patterned spiking, although quantitative comparisons of the patterns produced with experimental data have not been reported [26]. This previous model requires slow deterministic changes in model parameters to produce clustered patterns of spiking, whereas the model we propose here demonstrates that such slow changes are not necessary for the emergence of clustered spike firing. Nevertheless, it is possible that in entorhinal stellate neurons slow changes in ion channel states could further influence spike firing patterns in addition to the activity-dependent changes in spike probability that we describe here.

A conductance-based stochastic model [21] and a more abstract stochastic resonate-and-fire (SIF) model [44] have also been developed to account for the properties of stellate neurons. These models successfully account for the complex spectral properties of perithreshold fluctuations of membrane potential that are recorded experimentally and that are also generated by the stochastic model we describe here. It was previously suggested that a simplified, stochastic I_NaP_ is sufficient to produce patterened spiking [21] through random threshold crossings and spike omissions [8],[9]. However, the spiking patterns produced due to a stochastic I_NaP_ alone are nearly identical to a stochastic point process with a refractory period and thus do not provide a good match to the patterning observed experimentally (see Figure 4 in [21]). By contrast, the stochastic model we describe here produces more complex spike patterning that is a better match to the characteristics of clustered firing observed experimentally and not well described by a refractory Poisson process (Figure 6). The mechanism that we suggest for generation of clustered firing patterns also differs markedly from a recently proposed resonate-and-fire model that also reproduces clustered firing patterns of stellate cells [44]. The resonate and fire model explicitly states that sub-threshold resonance mechanisms are required to generate clustered spike patterns, whereas recent experimental studies clearly dissociate sub-threshold resonance from clustered spike firing patterns of stellate cells [18],[24]. Consistent with this data, the probabilistic model that we propose does not require sub-threshold resonance for generation of clustered spike firing and can provide a mechanistic explanation for dissociation of these two properties.

### Limitations of the Stochastic Model

There remain features of the firing patterns recorded experimentally from stellate neurons that are not well captured by any model proposed so far. A striking feature of some stellate neurons is a fairly regular intercluster interval even in the absence of coherent subthreshold oscillations (*e.g.* Figure S10). Our model stellate neuron, however, appears to exhibit more widely distributed intercluster intervals. One likely cause of this discrepancy is the simplification of the AHP current used in the model. Indeed, early results demonstrated that blockade of calcium entry can reduce the tendency of spiking to be clustered [15]. Since neuronal morphology can influence patterns of spike output [55], a further important limitation to the model that we propose here is that it is composed of only a single compartment. On the one hand, this could lead to an underestimate of the influence of stochastic gating, as in an extended dendritic structure fewer ion channels would contribute to ionic currents in any single compartment and thus the influence of stochastic channel gating on the membrane potential would be greater, as has been argued to be the case for thin axons [56]. On the other hand, comparison between our simulations and experimental data suggest that the magnitude of perithreshold oscillations and extent of spike clustering are comparable or perhaps larger in the model. Assessing the contribution of stochastic ion channel gating to the spatially distributed properties of stellate neurons will require future studies with more detailed computational models developed in parallel with more detailed electrical measurements from spatially distinct regions of the neuron. Nonetheless, the general principles that we establish here are likely to be robust to differences in morphology and although further morphological data may improve the similarity between our model data and the experimental data, simple models of neurons, neural circuits, and behavior can provide important functional insights in the absence of exhaustive detail [57].

### HCN Channels Support Patterned Spiking

Analysis of the stochastic model supports a key role for HCN channels in controlling the pattern of spike output from stellate neurons and suggests how the unique biophysical properties of HCN channels enable this role to be achieved. Thus, HCN channels active during the AHP fail to completely deactivate as the membrane potential returns to the steady-state (Figures 7 and 8). As a result, HCN channels briefly introduce a small bias current that substantially increases the probability of initiating a subsequent action potential (Figure 8). To the best of our knowledge this is a unique function of HCN channels that depends critically upon both their activation by membrane hyperpolarization and their deactivation kinetics (Figures 7, 8, and S9). Such an interaction, between a bias current introduced by a slowly gating ionic current with a small single channel conductance such as I_h_ and rapidly varying currents composed of ion channels with larger single channel conductances, may be a general mechanism by which neurons produce changes in firing properties that pattern action potential output. Importantly, under naturalistic stimulus conditions, patterned spiking in the stochastic model can still provide significant modifications to the response properties of stellate neurons (Figure 9). Several neuronal subtypes have been reported to display perithreshold oscillations of membrane potential [58],[59]. If intrinsic oscillations in other neurons also arise from stochastic channel gating, then patterned action potential firing driven by the interaction between multiple stochastic currents may also be a more general feature of neuronal spiking.

### Relevance of Stochastic Channel Gating to Activity *In Vivo*

The entorhinal cortex is the last stage at which cortical information is processed prior to entering the hippocampal formation. Stellate cells in layer II constitute a major excitatory projection to the dentate gyrus and may correspond to the recently discovered ‘grid cells’, which encode an animal's location in its environment through grid-like spatial firing fields [49],[60],[61]. While unreliable synaptic transmission is often considered as a noise source in neural circuits [6], less attention is usually given to the possible impact of stochastic ion channel fluctuations. Using stimulus parameters selected to obtain output firing properties similar to those recorded *in vivo*, we found that the presence of stochastically gating ion channels reliably increased the number of action potentials emitted with an ISI characteristic of intra-cluster intervals (Figure 9). This tendency depended on the stimulus statistics used, but is consistent with the peak in the *in vivo* ISI histograms around 100 ms and with our explanation for clustering as a transient increase in spike probability during the ∼70–150 ms following an action potential. Since the trains of synaptic stimuli used for these simulations have random statistics, these data support the idea that the effects of stochastic ion channel gating may in some conditions be superimposed on, rather than overwhelmed by, synaptic noise sources. Thus, stochastic ion channel gating may have to be accounted for in order to explain the firing of grid cells in behaving animals. However, further evaluation of this hypothesis will require much more information about the actual synaptic inputs received by grid cells. In addition, to better compare *in vitro* and *in vivo* data future studies will be required to establish whether *in vivo* data sets obtained from superficial layers of the MEC are indeed enriched for stellate neurons [49],[60].

We also attempted to predict the responses of wild-type and HCN1 knockout neurons to naturalistic stimuli. These simulations suggested that in the absence of changes to the input stimulus, stellate neurons lacking HCN1 will have an approximately 65% reduction in average firing rate (Figure 9). This reduced firing rate is characterized by an increase in the fraction of spikes emitted in high frequency bursts, or a sparsening of the response properties. There have been several suggestions that high frequency bursts convey unique information [62]–[64] about input stimuli and thus, this change could contribute to the enhancement of hippocampus-dependent learning in mice with deletion of HCN1 channels [28].

### Conclusion

Whereas initiation of action potentials in deterministic model neurons is a binary process with a clearly definable threshold, in more realistic neuronal models containing stochastically gating ion channels spike initiation is probablistic. Here we show that one general consequence of stochastic ion channel gating is that firing of an action potential can transiently modify the spike probability leading to the emergence of intrinsically generated patterns of spike output. In the case of the model we develop here, activation of HCN channels, during recovery from the action potential afterhyperpolarization, drives a brief increase in spike probability that leads to the emergence of clustered patterns of spike firing. As well as providing an account of both the resting and active integrative properties of stellate neurons in the medial entorhinal cortex, analysis of responses of this model to simulated *in vivo* synaptic inputs, suggests conditions in which stochastic ion channel gating might impact firing patterns of behaving animals. Thus, our results suggest a mechanism by which random changes in the conformation of small numbers of individual ion channel proteins could impact neural computations that underlie cognitive processes such as spatial navigation and memory.

## Materials and Methods

### Model Implementation

Modeling experiments were implemented in Matlab 7 (Natick, MA) using kinetic formalisms described in Text S1. The model has also been completely replicated in NEURON 5.9, but Matlab simulations were used for the data reported. The model cell was a sphere with a diameter of 50 µm and a specific capacitance of 1.67 µF/cm^2^ (to account for the lack of a dendritic arbor). The model included implementations of a fast, transient sodium current (NaT), a persistent sodium current (NaP), a delayed rectifier-type postssium current (Kdr), a fast inactivating A-type potassium current (KaF) and a slowly inactivating potassium current (KaS), a “calcium-activated” potassium current (KCa), a linear potassium leak (Kl) and a fast or slow hyperpolarization-activated current (Hf or Hs). Hf, Hs and KCa are implemented as two-state channels, which is sufficient to capture their dominant kinetics, although additional states would be required to more fully capture details of their gating. NaP, KaF, and KaS, were modeled with a cyclical four state inactivation model. NaT and Kdr currents were modeled according to the original Hodgkin-Huxley formalism with 5 and 8 states, respectively. The total current density of each channel was closely matched to existing data.

In order to model stochastic channels, it was assumed that the states obeyed a first order Markov-type probabilistic description [2]. To track channel populations in each state a random number was generated for each channel in a given state (a “particle”) at each time step (Δt). Assuming that the time step is sufficiently small the probability of a transition is equal to rate×Δt, with a transition occurring in the event that a random number, evenly distributed between 0 and 1 is less than rate×Δt. For particles with multiple possible transitions (*i.e.* multistate channels that have multiple transitions into and out of a given state), a unique transition was chosen using non-overlapping distributions of transition probabilities. Briefly, a uniformly distributed collection of random numbers between zero and one, thresholded by the value P (transition) will give *N*, the number of transitions that occur. In the case where multiple transitions are possible, we observe that a given “particle” can only undergo a single transition. We know from probability theory that:
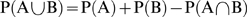
However, if there can only be a single transition then:
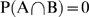
and thus,
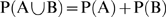



The probability that a given transition occurs is then the sum of the elementary probabilities. Dividing the probability space between 0 and 1 into bins of size P(A), P(B) and 1- P(A∪B), and placing random variables uniformly distributed between 0 and 1, gives the desired values for the number of transitions. This brute force method is similar to the simple Monte Carlo method described elsewhere [65] and to the method used elsewhere to model stochastic channels [9].

The time step used was 10 µs (corresponding to the approximate minimum dwell time of NaT) and numerical integration was accomplished using a 4th order Runge-Kutta method (most results were confirmed using the Backward Euler integration method). Simulations were run in Matlab and all analysis was completed using Igor Pro (Wavemetrics; Eugene, OR). A complete description of parameters used for the model currents and justification of parameters can be found in Text S1. Further, each channel was implemented as either stochastic or deterministic and it was ensured that in all cases the two solutions converged. For some simulations a partially stochastic model was used to speed simulation times and provide a good estimate of the fully stochastic model (data in Figures 4 and 9). This was justified by directly examining the contribution of each conductance (Figures S4 and S6).

### Definitions

Throughout the text we have made reference to a number of descriptions of the biophysical properties of the neuron that are elaborated upon here for clarity. The passive membrane properties we characterize are the resting membrane conductance and resting membrane potential. Typically these values are obtained by analyzing the response of the membrane potential to small current steps. By convention we assume that the state of the voltage-dependent currents is unaltered. The values are then obtained by application of Ohm's Law. However, during active states, when the neuron or model is depolarized away from its resting potential, the assumption that the underlying conductances are unaltered by small changes in injected current are generally less safe. At depolarized potentials we use a modified definition of the membrane conductance and consider the “effective” membrane conductance. Here we define the effective membrane conductance as the slope of the relationship between the membrane current and the membrane potential. This definition thus explicitly takes in to account the change in membrane conductance in response to a change in membrane voltage [2],[5].

### Analysis

All simulation data were analyzed in IGOR Pro (Wavemetrics) using both built-in analysis functions and custom written routines. Unless indicated otherwise mean values are ±standard error of the mean (SEM). Statistical tests were accomplished using Excel (Microsoft) and IGOR Pro (Wavemetrics).

#### Analysis of perithreshold fluctuations in membrane potential

To analyze the spectral properties of perithreshold fluctuations in membrane potential the built-in sonogram function of IGRO Pro was used to estimate the short-time fourier transform (STFT) of the membrane potential response to 20 s epochs of DC current injection to the model. We further used the fast Fourier transform (FFT) function to determine the spectral properties for the entire epoch and selected 1 s sub-epochs of the response. For the full 20 s analysis the FFT result was smoothed using a Savitzky-Golay algorithm (35 point) for improved display. Representative epochs were chosen from the central 10 s of data based upon the appearance of coherent oscillatory behavior and consistent with the changes in the power spectrum observed by analyzing all such brief epochs. To compare the amplitude of oscillations the mean of the integrated power spectra between 5–10 Hz was calculated for all epochs.

#### Analysis of spike patterning

To quantify the tendency for neurons to generate clustered patterns of spikes, we used previous ‘relaxed’ and ‘stringent’ definitions [18] for the data in Figure 4. Subsequently, we used a single intermediate definition (400 ms intercluster interval) to allow a single value to be reported where helpful. Thus, a cluster of spikes was defined as two or more consecutive spikes with interspike (intracluster) interval <250 ms, preceded and followed by silent periods (intercluster intervals) of duration >300 ms (relaxed), 400 ms (intermediate), or 500 (stringent) ms. We estimated the probability that a spike occurs as part of a cluster 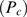 from the ratio of the number of spikes that occur within clusters to the total number of spikes. Data were binned according to the average firing rate (inverse of the mean interspike interval) for all spikes during the entire 150 s simulation or for each repetition of a 16 s trial (Figure 4D). Following the data in Figure 4D, all subsequent analysis used the single, intermediate definition of clustering.

#### Generation of P(s_t_|s_t0_) distributions

We collected all spike triggered membrane potential epochs for each simulation by thresholding the first derivative of the membrane potential. The spikes were aligned such that t = 0 at the threshold crossing, which was operationally defined as 10% of the maximum of the first derivative of the membrane potential. Using this ensemble of spike-triggered membrane potential epochs we detected spikes (using the same thresholded derivative) following the initial aligned spike to create a spike-triggered raster plot. The rasters were then binned (10 ms bins) and divided by the number of traces in the ensemble to generate a spike-triggered spike probability distribution as a function of time, *t*, following the time of the aligned spike, *t_0_*, or “P(s_t_|s_t0_)”.

#### Estimate of passive membrane properties

Simulations of responses to current steps (amplitude±5pA; duration 5 s) were run to estimate passive parameters of the stellate models. Input resistance (R_i_) was defined as the ratio of the steady state voltage in response to positive (“+”) or negative (“−”) current injection to the resting potential. Monoexponential fits to the initial voltage response were used to obtain the membrane time constant (τ_m_). The sag ratio is calculated as the ratio of the peak instantaneous voltage difference divided by the steady-state voltage difference for the negative current injection.

### Experimental Data from *In Vivo* Recordings

Analysis of *in vivo* recordings of cortical neurons from the superficial layers of the medial entorhinal cortex was based upon data obtained from: http://commonweb.ntnu.no/cbm/moser/gridcell.

### Synaptic Stimulation

For Figure 9 we attempted to provide a general, readily parameterized model of synaptic drive that might occur *in vivo*. Because we used a single compartment model, appropriately scaled current stimulation can be equivalent to conductance-based stimuli [66]. Further, in order to provide a readily parameterized stimulus to explore the space of possible responses we chose to use colored white noise. Again, over the range of frequencies where the impedance of the cell membrane is maximal, random barrages of synaptic input show approximately white stimulus statistics [66]. For our stimulus we thus create a broadband, white noise stimulus that was bandlimited to 50 Hz. The standard deviation and DC offset of the current stimulus were scaled according to the parameters in Figure 9A and 9B and applied directly to the model.

The ISI histogram of responses to the broadband stimulus was not as broad as for the *in vivo* experimental data. Examining the experimental data revealed that this was primarily due to a lack of high-frequency (>50 Hz) bursting in the model. We made the assumption that occasional changes in stimulus statistics could give rise to this high frequency bursting. By examining the approximate length of such periods we determined that ∼200 ms long changes in stimulus statistics were consistent with the experimental data. We assumed a Poisson distribution for the duration of these epochs of high frequency activity. We chose an interval between the high frequency epochs that gave an approximately correct balance in the ISI distribution (mean = 1 s; Poisson distributed). Finally, the amplitude of the changes in the DC component and standard deviation were taken from the survey of parameter space to match the central peak of the bursting ISIs (see Figure 9).

## Supporting Information

Figure S1 Stochastic gating can produce substantial channel noise
(0.70 MB PDF)

Figure S2 Membrane impedance determines the increased membrane potential fluctuations in the HCN1 knock-out model
(3.38 MB PDF)

Figure S3 I_h_ is not required for perithreshold oscillations
(2.78 MB PDF)

Figure S4 Necessity and sufficiency of stochastic conductances
(4.69 MB PDF)

Figure S5 Spiking properties of the deterministic model
(0.14 MB PDF)

Figure S6 Partially stochastic model does not significantly differ from completely stochastic model
(0.19 MB PDF)

Figure S7 Voltage threshold for spike initiation is not correlated with ISI
(0.08 MB PDF)

Figure S8 Determining the critical point for spike initiation
(0.34 MB PDF)

Figure S9 A wide range of HCN kinetics are sufficient for AHP enhancement
(0.58 MB PDF)

Figure S10 A regularly spiking MEC stellate neuron
(0.80 MB PDF)

Text S1 Components of the stellate model
(0.18 MB PDF)

## References

[1] Neher E, Sakmann B (1976). Single-channel currents recorded from membrane of denervated frog
muscle fibres.. Nature.

[2] Hille B (2001). Ion Channels of Excitable Membranes.

[3] Hodgkin AL, Huxley AF (1952). A quantitative description of membrane current and its
application to conduction and excitation in nerve.. J Physiol.

[4] Migliore M, Shepherd GM (2002). Emerging rules for the distributions of active dendritic
conductances.. Nat Rev Neurosci.

[5] Koch C (1999). Biophysics of Computation: Information Processing in Single Neurons.

[6] White JA, Rubinstein JT, Kay AR (2000). Channel noise in neurons.. Trends Neurosci.

[7] Waters J, Helmchen F (2006). Background synaptic activity is sparse in neocortex.. J Neurosci.

[8] Chow CC, White JA (1996). Spontaneous action potentials due to channel fluctuations.. Biophys J.

[9] Schneidman E, Freedman B, Segev I (1998). Ion channel stochasticity may be critical in determining the
reliability and precision of spike timing.. Neural Comput.

[10] Diba K, Koch C, Segev I (2006). Spike propagation in dendrites with stochastic ion channels.. J Comput Neurosci.

[11] Jacobson GA, Diba K, Yaron-Jakoubovitch A, Oz Y, Koch C (2005). Subthreshold voltage noise of rat neocortical pyramidal neurones.. J Physiol.

[12] Dolorfo CL, Amaral DG (1998). Entorhinal cortex of the rat: organization of intrinsic
connections.. J Comp Neurol.

[13] Witter MP, Amaral DG (1991). Entorhinal cortex of the monkey: V. Projections to the dentate
gyrus, hippocampus, and subicular complex.. J Comp Neurol.

[14] Alonso A, Klink R (1993). Differential electroresponsiveness of stellate and pyramidal-like
cells of medial entorhinal cortex layer II.. J Neurophysiol.

[15] Klink R, Alonso A (1993). Ionic mechanisms for the subthreshold oscillations and
differential electroresponsiveness of medial entorhinal cortex layer II
neurons.. J Neurophysiol.

[16] Alonso A, Llinás RR (1989). Subthreshold Na^+^-dependent theta-like
rhythmicity in stellate cells of entorhinal cortex layer II.. Nature.

[17] Dickson CT, Magistretti J, Shalinsky M, Hamam B, Alonso A (2000). Oscillatory activity in entorhinal neurons and circuits.
Mechanisms and function.. Ann N Y Acad Sci.

[18] Nolan MF, Dudman JT, Dodson PD, Santoro B (2007). HCN1 channels control resting and active integrative properties
of stellate cells from layer II of the entorhinal cortex.. J Neurosci.

[19] Buzsáki G (2002). Theta oscillations in the hippocampus.. Neuron.

[20] Dorval AD, White JA (2005). Channel noise is essential for perithreshold oscillations in
entorhinal stellate neurons.. J Neurosci.

[21] White JA, Klink R, Alonso A, Kay AR (1998). Noise from voltage-gated ion channels may influence neuronal
dynamics in the entorhinal cortex.. J Neurophysiol.

[22] Haas JS, Dorval AD, White JA (2007). Contributions of I_h_ to feature selectivity in layer II
stellate cells of the entorhinal cortex.. J Comput Neurosci.

[23] Haas JS, White JA (2002). Frequency selectivity of layer II stellate cells in the medial
entorhinal cortex.. J Neurophysiol.

[24] Fernandez FR, White JA (2008). Artificial synaptic conductances reduce subthreshold oscillations
and periodic firing in stellate cells of the entorhinal cortex.. J Neurosci.

[25] Dickson CT, Magistretti J, Shalinsky MH, Fransén E, Hasselmo ME (2000). Properties and role of I_h_ in the pacing of
subthreshold oscillations in entorhinal cortex layer II neurons.. J Neurophysiol.

[26] Fransén E, Alonso AA, Dickson CT, Magistretti J, Hasselmo ME (2004). Ionic mechanisms in the generation of subthreshold oscillations
and action potential clustering in entorhinal layer II stellate neurons.. Hippocampus.

[27] Nolan MF, Malleret G, Lee KH, Gibbs E, Dudman JT (2003). The hyperpolarization-activated HCN1 channel is important for
motor learning and neuronal integration by cerebellar Purkinje cells.. Cell.

[28] Nolan MF, Malleret G, Dudman JT, Buhl DL, Santoro B (2004). A behavioral role for dendritic integration: HCN1 channels
constrain spatial memory and plasticity at inputs to distal dendrites of CA1
pyramidal neurons.. Cell.

[29] Magee J (1999). Dendritic I_h_ normalizes temporal summation in
hippocampal CA1 neurons.. Nat Neurosci.

[30] Magee JC (1998). Dendritic hyperpolarization-activated currents modify the
integrative properties of hippocampal CA1 pyramidal neurons.. J Neurosci.

[31] Rosenkranz JA, Johnston D (2006). Dopaminergic regulation of neuronal excitability through
modulation of I_h_ in layer V entorhinal cortex.. J Neurosci.

[32] Kole MHP, Hallermann S, Stuart GJ (2006). Single I_h_ channels in pyramidal neuron dendrites:
properties, distribution, and impact on action potential output.. J Neurosci.

[33] Strauss U, Kole MHP, Bräuer AU, Pahnke J, Bajorat R (2004). An impaired neocortical I_h_ is associated with enhanced
excitability and absence epilepsy.. Eur J Neurosci.

[34] Otmakhova NA, Lisman JE (2004). Contribution of I_h_ and GABA_B_ to
synaptically induced afterhyperpolarizations in CA1: a brake on the NMDA
response.. J Neurophysiol.

[35] Berger T, Larkum ME, Lüscher HR (2001). High I_h_ channel density in the distal apical dendrite
of layer V pyramidal cells increases bidirectional attenuation of EPSPs.. J Neurophysiol.

[36] Southan AP, Morris NP, Stephens GJ, Robertson B (2000). Hyperpolarization-activated currents in presynaptic terminals of
mouse cerebellar basket cells.. J Physiol.

[37] Williams SR, Christensen SR, Stuart GJ, Hausser M (2002). Membrane potential bistability is controlled by the
hyperpolarization-activated current I_H_ in rat cerebellar Purkinje
neurons in vitro.. J Physiol.

[38] Tsay D, Dudman JT, Siegelbaum SA (2007). HCN1 channels constrain synaptically evoked
Ca^2+^ spikes in distal dendrites of CA1 pyramidal
neurons.. Neuron.

[39] Hasselmo ME, Fransen E, Dickson C, Alonso AA (2000). Computational modeling of entorhinal cortex.. Ann N Y Acad Sci.

[40] Conti F, Wanke E (1975). Channel noise in nerve membranes and lipid bilayers.. Q Rev Biophys.

[41] Cannon RC, D'Alessandro G (2006). The ion channel inverse problem: neuroinformatics meets
biophysics.. PLoS Comput Biol.

[42] Erchova I, Kreck G, Heinemann U, Herz AV (2004). Dynamics of rat entorhinal cortex layer II and III cells:
characteristics of membrane potential resonance at rest predict oscillation
properties near threshold.. J Physiol.

[43] Giocomo LM, Zilli EA, Fransen E, Hasselmo ME (2007). Temporal frequency of subthreshold oscillations scales with
entorhinal grid cell field spacing.. Science.

[44] Engel T, Schimansky-Geier L, Herz A, Schreiber S, Erchova IA (2008). Subthreshold membrane-potential resonances shape spike-train
patterns in the entorhinal cortex.. J Neurophysiol.

[45] Perkel DH, Gerstein GL, Moore GP (1967). Neuronal spike trains and stochastic point processes. I. The
single spike train.. Biophys J.

[46] Harris-Warrick RM (2002). Voltage-sensitive ion channels in rhythmic motor systems.. Curr Opin Neurobiol.

[47] Izhikevich (2000). Neural excitability, spiking and bursting.. Int J Bifurcat Chaos.

[48] Kole MH, Ilschner SU, Kampa BM, Williams SR, Ruben PC (2008). Action potential generation requires a high sodium channel
density in the axon initial segment.. Nat Neurosci.

[49] Hafting T, Fyhn M, Molden S, Moser M-B, Moser EI (2005). Microstructure of a spatial map in the entorhinal cortex.. Nature.

[50] Dorval AD (2008). Probability distributions of the logarithm of inter-spike
intervals yield accurate entropy estimates from small datasets.. J Neurosci Methods.

[51] Buzsaki G, Draguhn A (2004). Neuronal oscillations in cortical networks.. Science.

[52] Turrigiano GG, Nelson SB (2000). Hebb and homeostasis in neuronal plasticity.. Curr Opin Neurobiol.

[53] Hopfield JJ, Tank DW (1986). Computing with neural circuits: a model.. Science.

[54] Shadlen MN, Newsome WT (1998). The variable discharge of cortical neurons: implications for
connectivity, computation, and information coding.. J Neurosci.

[55] Mainen ZF, Sejnowski TJ (1996). Influence of dendritic structure on firing pattern in model
neocortical neurons.. Nature.

[56] Faisal AA, Laughlin SB (2007). Stochastic simulations on the reliability of action potential
propagation in thin axons.. PLoS Comput Biol.

[57] Dayan P, Abbott LF (2001). Theoretical Neuroscience: Computational and Mathematical Modeling of
Neural Systems.

[58] Zhu JJ, Lytton WW, Xue JT, Uhlrich DJ (1999). An intrinsic oscillation in interneurons of the rat lateral
geniculate nucleus.. J Neurophysiol.

[59] Strata F (1998). Intrinsic oscillations in CA3 hippocampal pyramids: physiological
relevance to theta rhythm generation.. Hippocampus.

[60] Sargolini F, Fyhn M, Hafting T, McNaughton BL, Witter MP (2006). Conjunctive representation of position, direction, and velocity
in entorhinal cortex.. Science.

[61] Fyhn M, Molden S, Witter MP, Moser EI, Moser M-B (2004). Spatial representation in the entorhinal cortex.. Science.

[62] Izhikevich EM, Desai NS, Walcott EC, Hoppensteadt FC (2003). Bursts as a unit of neural information: selective communication
via resonance.. Trends Neurosci.

[63] Metzner W, Koch C, Wessel R, Gabbiani F (1998). Feature extraction by burst-like spike patterns in multiple
sensory maps.. J Neurosci.

[64] Lisman JE (1997). Bursts as a unit of neural information: making unreliable
synapses reliable.. Trends Neurosci.

[65] Press W, Teukolsky S, Vetterling W, Flannery B (1992). Numerical Recipes in C: The Art of Scientific Computing.

[66] Destexhe A, Rudolph M (2004). Extracting information from the power spectrum of synaptic noise.. J Comput Neurosci.

