# Supplementary material for: Stochastically Gating Ion Channels Enable Patterned Spike Firing through Activity-Dependent Modulation of Spike Probability
Source: PLoS Comput Biol. 2009 Feb 13;5(2):e1000290. doi: 10.1371/journal.pcbi.1000290 (PMC2631146; doi:10.1371/journal.pcbi.1000290)
Supplement: Figure S1 — Stochastic gating can produce substantial channel noise (0.70 MB PDF) [file pcbi.1000290.s001.pdf]

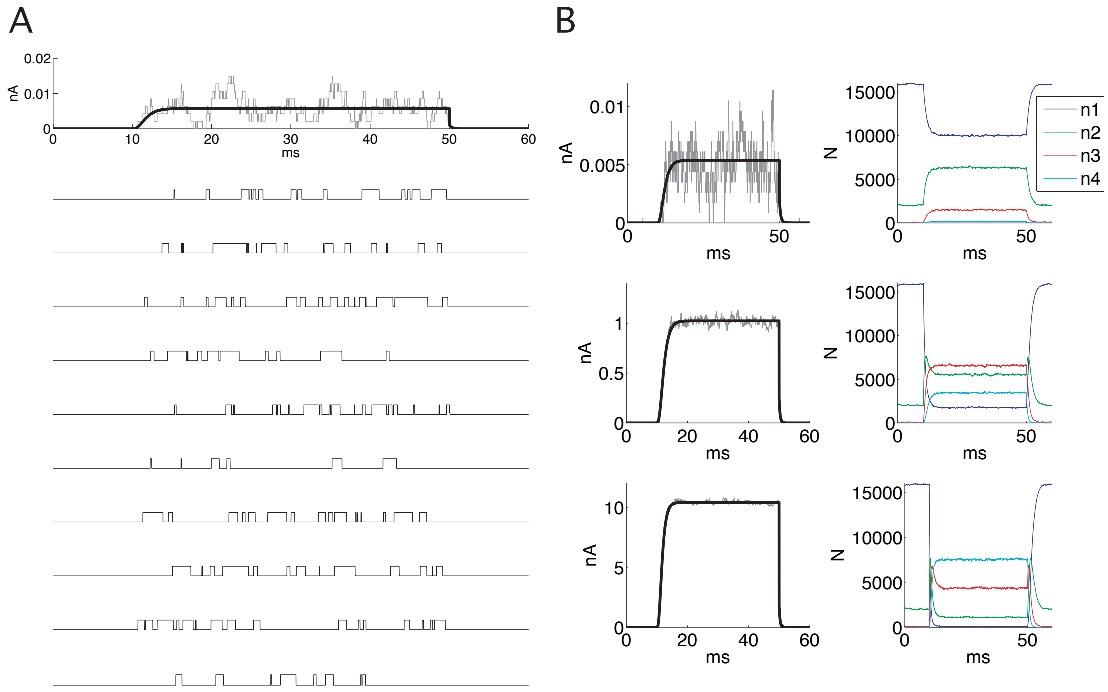

Figure S 1: **Stochastic gating can produce substantial channel noise** Example voltage-clamp simulation of the delayed rectifier potassium current (Kdr) used in the complete stochastic stellate model. (A) A voltage clamp simulation of the deterministic (solid black) and stochastic (thin black line) models of the current flow through 10 Kdr channels in response to a step depolarization. Lower traces are the microscopic currents flowing through each individual Kdr channel that sum to produce the thin trace in the top plot. (B) Voltage clamp simulations with the entire complement of Kdr channels used in the complete model. Current responses for the deterministic (solid black) and stochastic (thin black line) models are shown in the plots at left in response to steps from a holding potential of -70 mV to command potentials of -60 (top), -50 (middle) and -40 (bottom) mV. The corresponding occupancies of the closed states are shown at right. Note the signal to noise is proportional to the number of channels near the open state, not the total number of channels.
