# Supplementary material for: Stochastically Gating Ion Channels Enable Patterned Spike Firing through Activity-Dependent Modulation of Spike Probability
Source: PLoS Comput Biol. 2009 Feb 13;5(2):e1000290. doi: 10.1371/journal.pcbi.1000290 (PMC2631146; doi:10.1371/journal.pcbi.1000290)
Supplement: Figure S2 — Membrane impedance determines the increased membrane potential fluctuations in the HCN1 knock-out model (3.38 MB PDF) [file pcbi.1000290.s002.pdf]

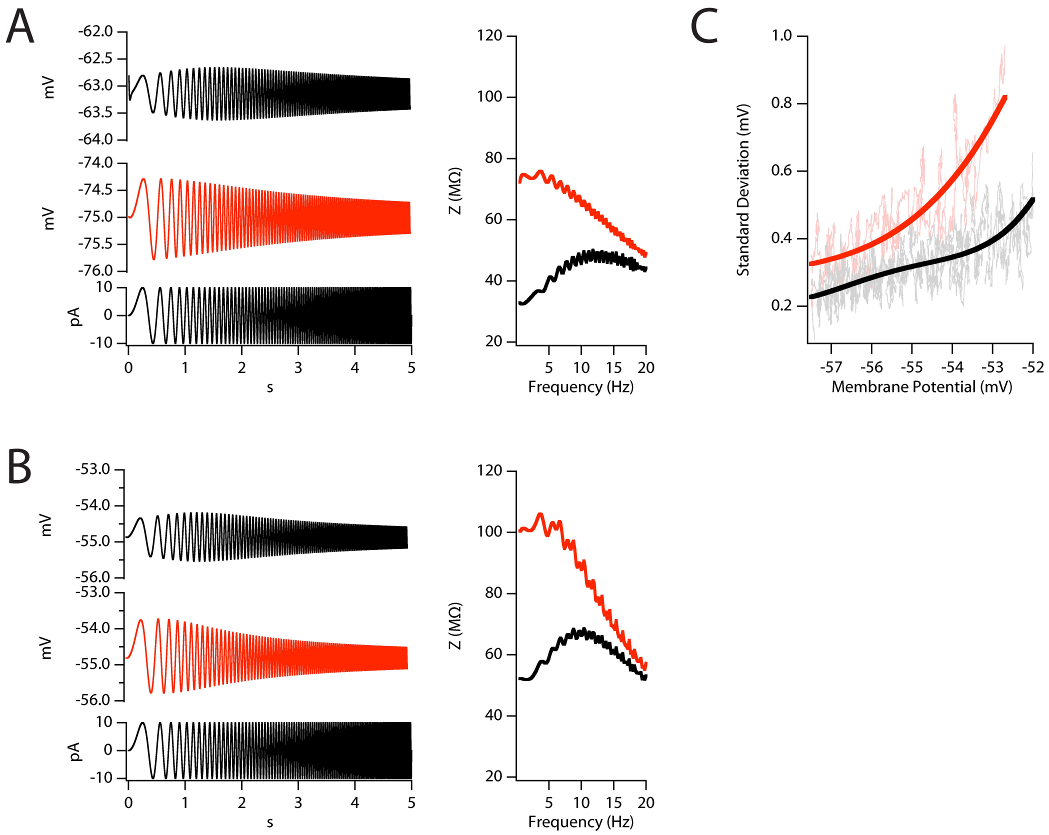

Figure S2: **Membrane impedance determines the increased membrane potential fluctuations in the HCN1 knock-out model** The membrane impedance (right plots) was determined at the resting potential (A) and a depolarized potential (B) by injecting sinusoidal currents of linearly increasing frequency (bottom graphs) to the wild-type (black) and HCN1 knockout (red) models. (C) Using the data from ramp currents (see Figure 3) the standard deviation of trial to trial variation in membrane potential is plotted against the mean membrane potential for each time point in the wild-type (black) and HCN1 knockout (red) simulations. Noisy traces were fit with a 5th order polynomial to show the trend and the steeper increase in membrane potential fluctuations in the knock-out model due to the increased membrane impedance.
