# Supplementary material for: Stochastically Gating Ion Channels Enable Patterned Spike Firing through Activity-Dependent Modulation of Spike Probability
Source: PLoS Comput Biol. 2009 Feb 13;5(2):e1000290. doi: 10.1371/journal.pcbi.1000290 (PMC2631146; doi:10.1371/journal.pcbi.1000290)
Supplement: Figure S3 — Ih is not required for perithreshold oscillations (2.78 MB PDF) [file pcbi.1000290.s003.pdf]

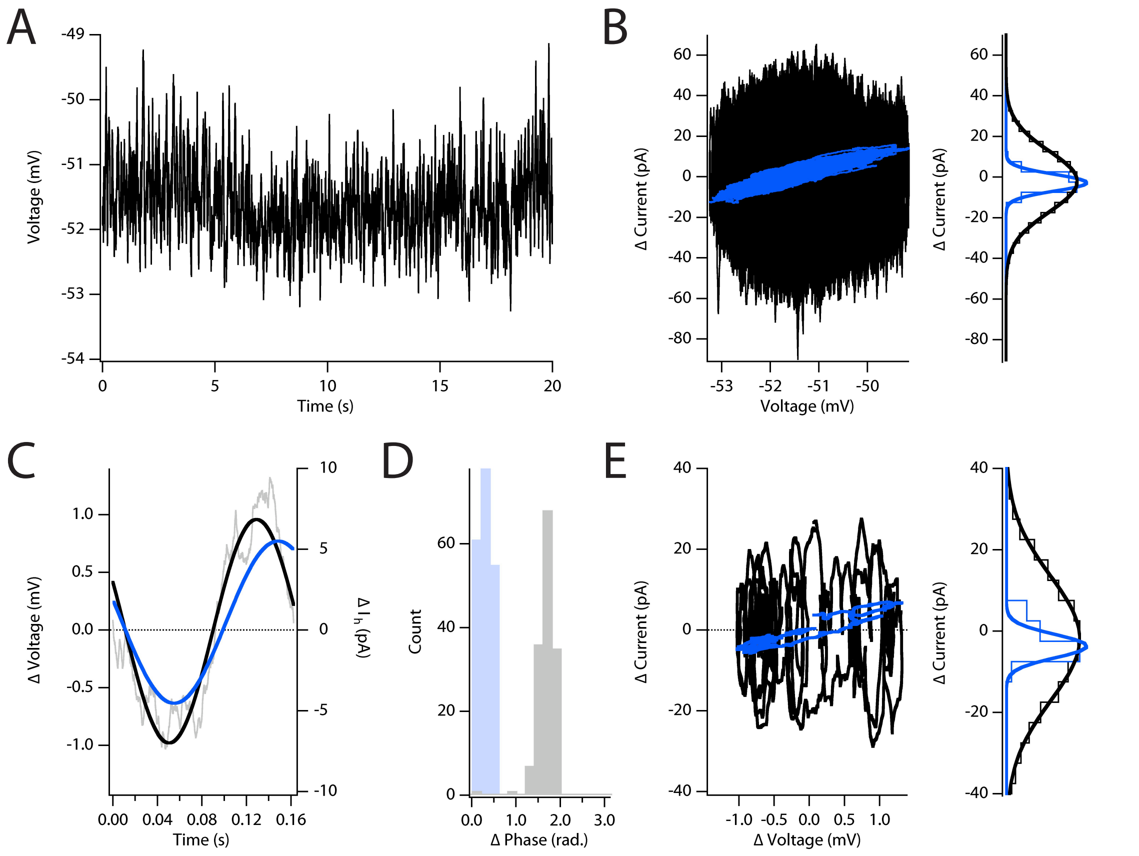

Figure S3: **I_h is not required for perithreshold oscillations** To determine whether I_h was an important determinant of the perithreshold membrane potential fluctuations the currents underlying a long (20 s) simulation were examined. (A) The membrane potential showed prominent fluctuations. (B) A phase plot of the underlying currents was generated by separating the net ionic current into an I_h (blue) component and a residual component (black) composed of the other 7 currents in the model. The mean is subtracted so that the currents can be overlaid for comparison. (B, right) The distribution of instantaneous current amplitudes in the phase plot. (C) The membrane potential during a single oscillation period is overlaid with the corresponding HCN current. Solid lines are sinusoidal fits. (D) The instantaneous phases of the membrane potential, I_h component and residual component fits were determined using the Hilbert transform. A histogram of the absolute values of the phase difference between the membrane potential and the I_h component (blue) and residual component (black). (E) Underlying ionic currents during the membrane potential epoch shown in D. Currents and plots as in B. Currents were smoothed for easier visualization. (E, right) The distribution of instantaneous current amplitudes for the epoch shown in D.
