# Supplementary material for: Stochastically Gating Ion Channels Enable Patterned Spike Firing through Activity-Dependent Modulation of Spike Probability
Source: PLoS Comput Biol. 2009 Feb 13;5(2):e1000290. doi: 10.1371/journal.pcbi.1000290 (PMC2631146; doi:10.1371/journal.pcbi.1000290)
Supplement: Figure S4 — Necessity and sufficiency of stochastic conductances (4.69 MB PDF) [file pcbi.1000290.s004.pdf]

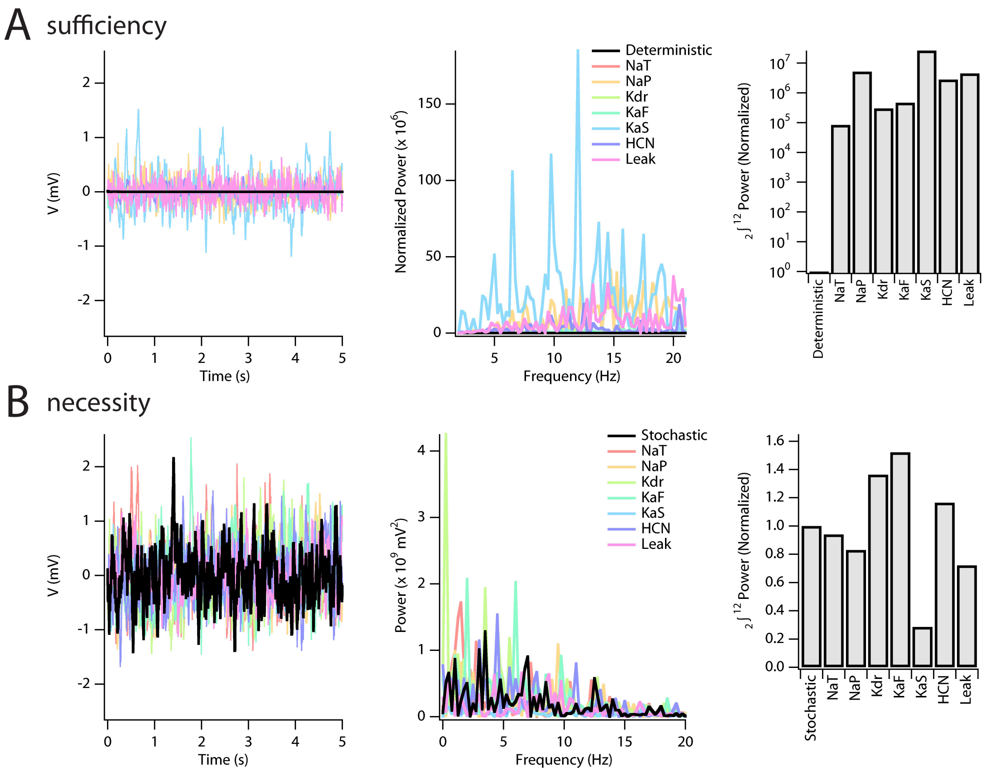

Figure S4: **Necessity and sufficiency of stochastic conductances** A series of simulations were run where each individual conductance was toggled between a stochastic and deterministic implementation. (A, "sufficiency") In the "sufficiency" experiments a completely deterministic model (black lines) was compared with a model in which each conductance was implemented as a stochastic conductance individually. (Left panel) A plot of membrane potential for each simulation. (Center panel) A plot of the power spectra of the membrane potential for the entire 5 s of simulation. (Right panel) To examine the role of each channel the power spectra were integrated between 2 and 12 Hz and normalized by the deterministic model. (B, "necessity") Analogous to the simulations in A, a completely stochastic model was compared with simuations in which individual conductances were modeled as deterministic conductances. Corresponding panels are the same as in A.
