# Supplementary material for: Stochastically Gating Ion Channels Enable Patterned Spike Firing through Activity-Dependent Modulation of Spike Probability
Source: PLoS Comput Biol. 2009 Feb 13;5(2):e1000290. doi: 10.1371/journal.pcbi.1000290 (PMC2631146; doi:10.1371/journal.pcbi.1000290)
Supplement: Figure S5 — Spiking properties of the deterministic model (0.14 MB PDF) [file pcbi.1000290.s005.pdf]

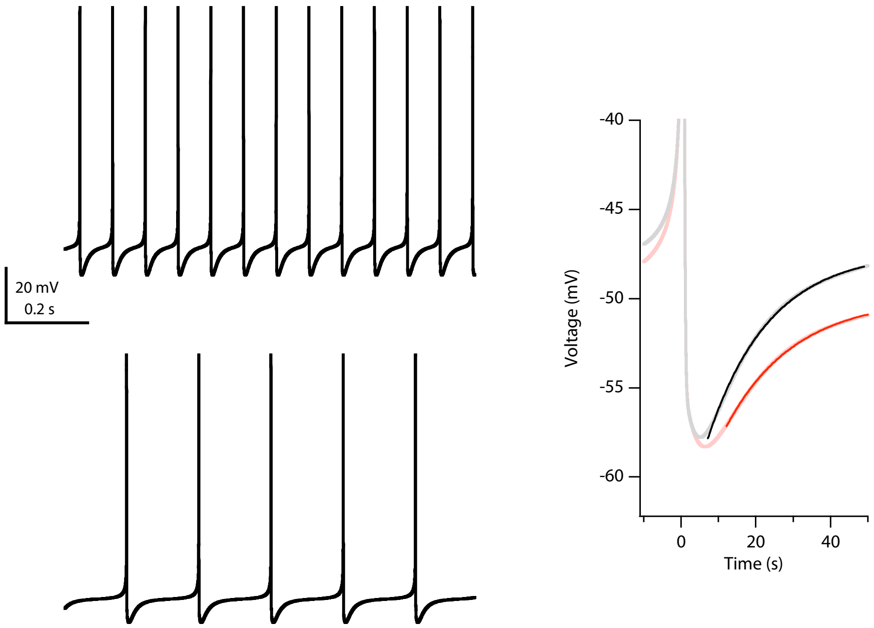

Figure S5: **Spiking properties of the deterministic model** Spiking responses in response to threshold current injection into the wild-type (upper trace) and HCN1 knock-out (lower trace) deterministic models. Spikes were aligned and averaged (right plot) for the wild-type (black) and HCN1 knock-out (red) simulations. Note the difference in AHP recovery (higlighted with exponential fit) between the models.
