# Supplementary material for: Stochastically Gating Ion Channels Enable Patterned Spike Firing through Activity-Dependent Modulation of Spike Probability
Source: PLoS Comput Biol. 2009 Feb 13;5(2):e1000290. doi: 10.1371/journal.pcbi.1000290 (PMC2631146; doi:10.1371/journal.pcbi.1000290)
Supplement: Figure S6 — Partially stochastic model does not significantly differ from completely stochastic model (0.19 MB PDF) [file pcbi.1000290.s006.pdf]

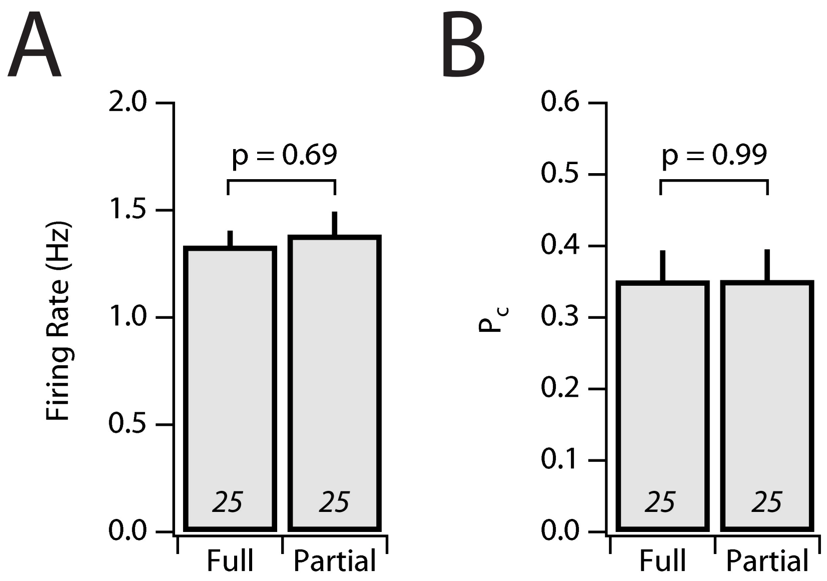

Figure S6: **Partially stochastic model does not significantly differ from completely stochastic model** Two sets (N=25) of simulations of the stochastic model were run with an injected DC current. A completely stochastic model ("Full") was compared with a model in which the HCN current, NaT and Kdr currents were deterministic ("Partial"). We found no significant difference in the resultant firing rate (A) or probability of clustering (P_c; B).
