# Supplementary material for: Stochastically Gating Ion Channels Enable Patterned Spike Firing through Activity-Dependent Modulation of Spike Probability
Source: PLoS Comput Biol. 2009 Feb 13;5(2):e1000290. doi: 10.1371/journal.pcbi.1000290 (PMC2631146; doi:10.1371/journal.pcbi.1000290)
Supplement: Figure S7 — Voltage threshold for spike initiation is not correlated with ISI (0.08 MB PDF) [file pcbi.1000290.s007.pdf]

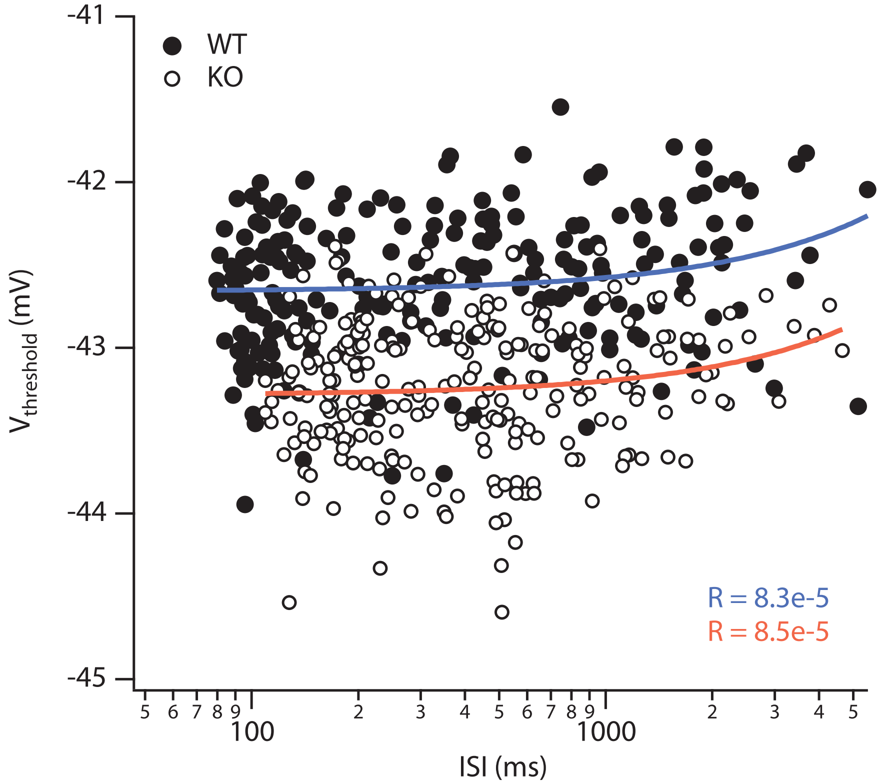

Figure S7: **Voltage threshold for spike initiation is not correlated with ISI** The voltage threshold for spike initiation was determined by thresholding the first-derivative of the membrane potential. The lowest threshold that did not yield false-positives was determined and applied to long simulations of the stochastic models. The membrane potential at which the detection threshold was crossed in the wild-type (closed circles) and HCN1 knock-out (open circles) simulations was plotted against the succeeding ISI. Linear fits showed no significant correlation between threshold membrane potential and ISI. Slopes indicated on graph.
