# Supplementary material for: Stochastically Gating Ion Channels Enable Patterned Spike Firing through Activity-Dependent Modulation of Spike Probability
Source: PLoS Comput Biol. 2009 Feb 13;5(2):e1000290. doi: 10.1371/journal.pcbi.1000290 (PMC2631146; doi:10.1371/journal.pcbi.1000290)
Supplement: Figure S8 — Determining the critical point for spike initiation (0.34 MB PDF) [file pcbi.1000290.s008.pdf]

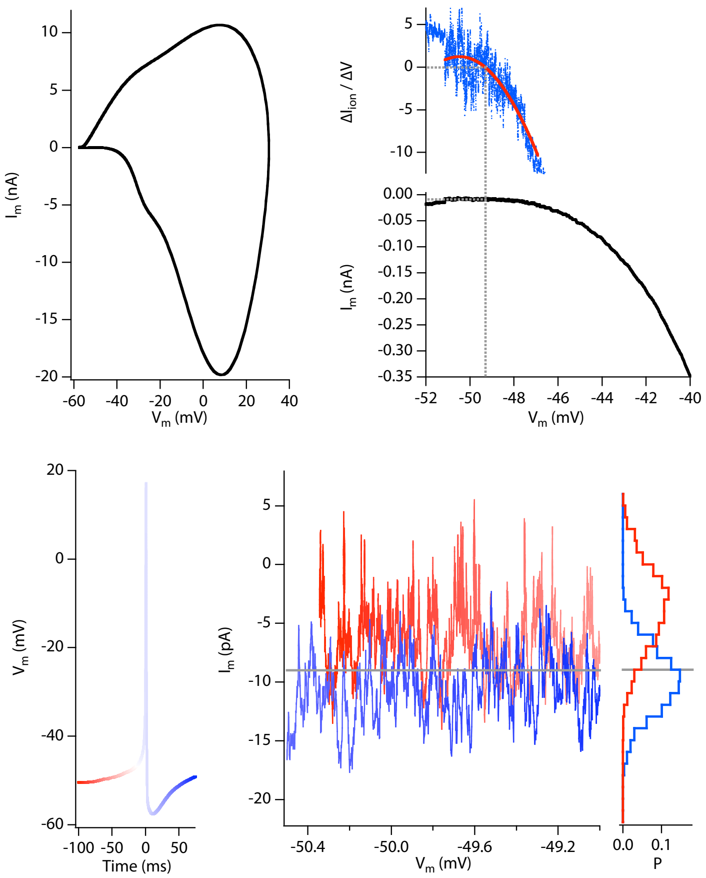

Figure S8: **Determining the critical point for spike initiation** (Upper left) A plot of the relationship between net membrane current and membrane potential was generated from all spikes in a long simulation of the wild-type stochastic model. (Upper right) The instantaneous derivative of the membrane current with respect to membrane potential (blue dots) was generated and fit with a log normal function (red line). Dotted lines indicate the membrane potential at which the slope of the membrane current with respect to potential becomes negative. (Lower left) Average membrane potential for the aligned spikes. Line color of the membrane potential varies as a function of time to indicate the time difference between membrane current epochs shown in phase plot at right. Probability densities for instantaneous current amplitudes are plotted at right revealing an inward shift in the net membrane current at the threshold membrane potential following a spike relative.
