# Supplementary material for: Stochastically Gating Ion Channels Enable Patterned Spike Firing through Activity-Dependent Modulation of Spike Probability
Source: PLoS Comput Biol. 2009 Feb 13;5(2):e1000290. doi: 10.1371/journal.pcbi.1000290 (PMC2631146; doi:10.1371/journal.pcbi.1000290)
Supplement: Figure S9 — A wide range of HCN kinetics are sufficient for AHP enhancement (0.58 MB PDF) [file pcbi.1000290.s009.pdf]

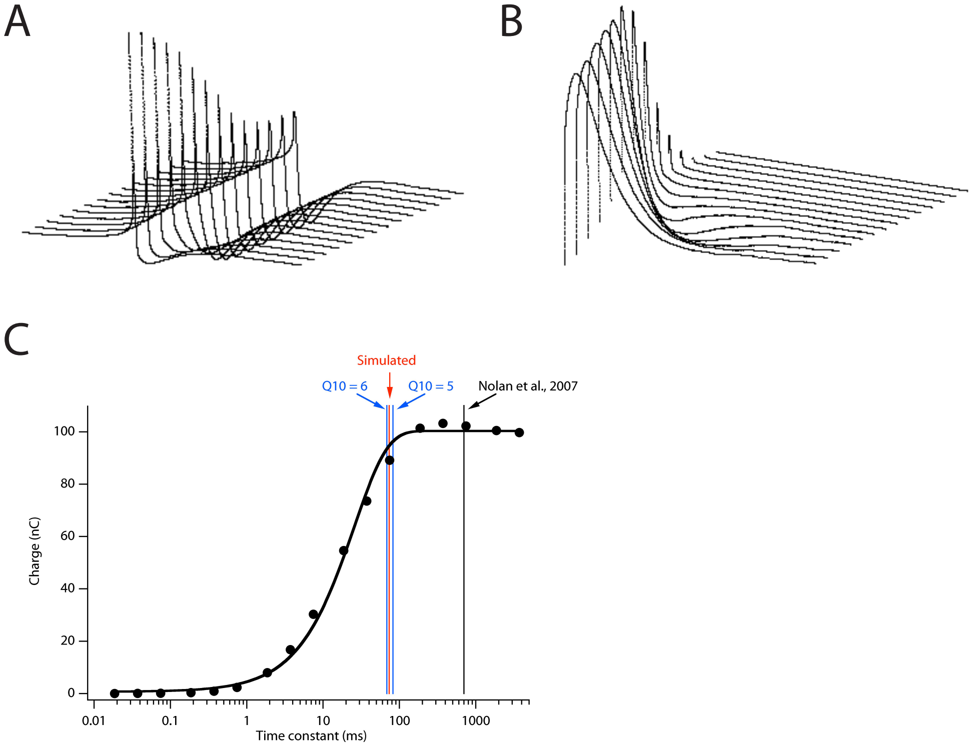

Figure S9: **A wide range of HCN kinetics are sufficient for AHP enhancement** (A) Example I_h currents recorded following a voltage-clamp simulation of the action potential waveform (as in FIgure 8) for a range of HCN channel kinetics. (B) Corresponding plots of I_{obs} - I_{ss} for the currents in A (again see Figure 8 in the main text for analysis details). (C) Total excess charge delivered from I_{obs} - I_{ss} is plotted for a range of HCN channels kinetics. The activation time constant at -70 mV is used to reflect channel kinetics. For comparison experimental values obtained at room temperature and estimates of corresponding values with assuming different Q10 values are indicated on the plot. Red line ("Simulated") indicates the value used in the WT model throughout the paper.
