# Supplementary material for: Stochastically Gating Ion Channels Enable Patterned Spike Firing through Activity-Dependent Modulation of Spike Probability
Source: PLoS Comput Biol. 2009 Feb 13;5(2):e1000290. doi: 10.1371/journal.pcbi.1000290 (PMC2631146; doi:10.1371/journal.pcbi.1000290)
Supplement: Figure S10 — A regularly spiking MEC stellate neuron (0.80 MB PDF) [file pcbi.1000290.s010.pdf]

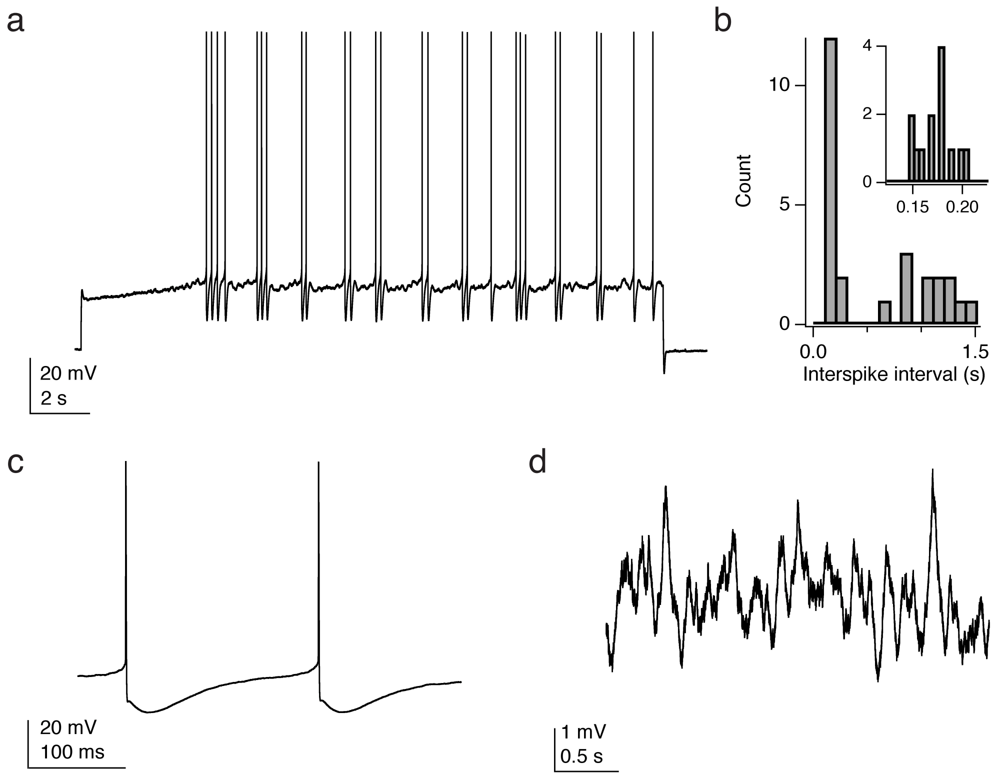

Figure S10: **A regularly spiking MEC stellate neuron** The key integrative properties of stellate neurons are revealed by long depolarizing current steps. (**a**) 20 s long injection of positive current causes a slow depolarization and stable spiking with little rate accomodation. Spikes are characteristically clustered in short, low frequency bursts of 2–5 action potentials. (**b**) For this particular cell interspike interval histogram reveals a clear bimodal distribution corresponding to clustered spiking. Note the wide distribution of both intracluster intervals (inset) and intercluster intervals. Intracluster intervals were well-fit by a gaussian centered at 170 ms with a width of 32 ms. (**c**) Individual action potentials show characteristic deep and slow afterhyperpolarizations. This short cluster reveals the low frequency of the burst. (**d**) A depolarizing current step to potentials just below threshold reveals prominent membrane potential fluctuations. These fluctuations have significant frequency components in the theta range (4–12 Hz), however the frequency, amplitude and phase are highly variable. It should be noted that this cell shows rather exemplary clustering, a property that is highly variable between neurons.
