# Supplementary material for: Stochastically Gating Ion Channels Enable Patterned Spike Firing through Activity-Dependent Modulation of Spike Probability
Source: PLoS Comput Biol. 2009 Feb 13;5(2):e1000290. doi: 10.1371/journal.pcbi.1000290 (PMC2631146; doi:10.1371/journal.pcbi.1000290)
Supplement: Text S1 — Components of the stellate model (0.18 MB PDF) [file pcbi.1000290.s011.pdf]

Supplementary Materials

December 27, 2008

Components of the stellate model

Properties of the model were determined using a variety of sources. The transient sodium current (NaT) and the delayed rectifier (Kdr) were modified from previous kinetic schemes (Hoffman DA, Magee JC, Colbert CM, Johnston D (1997) Nature 387: 869-875.) to give a threshold, amplitude and time course approximating action potentials recorded in: Nolan MF, Dudman JT, Dodson PD, Santoro B (2007) J Neurosci 27(46): 12440-12451. Properties and densities of the persistent sodium current were taken from: Magistretti J, Alonso A (1999) J Gen Physiol 114: 491-509. Single channel conductances for potassium currents were based upon: Chen X, Johnston D (2004) J Physiol 559(Pt 1): 187-203. Gating properties and single channel conductance of the leak current were derived from: Bockenhauer D, Zilberberg N, Goldstein SA (2001) Nat Neurosci. 4(5):486-91. The AHP current was modeled as a spike dependent current with a small single channel conductance similar to SK channels: Bond CT, Herson PS, Strassmaier T, Hammond R, Stackman R et al. (2004) J Neurosci 24: 5301-5306. HCN current kinetics and density used a simplified 2-state model based upon data from: Nolan MF, Dudman JT, Dodson PD, Santoro B (2007) J Neurosci 27(46): 12440-12451. The small single channel conductance was based upon classic measurements by DiFrancesco and colleagues (see Robinson RB, Siegelbaum SA (2003) Annu Rev Physiol 65: 453-480) and more recent measurements: Kole MHP, Hallermann S, Stuart GJ (2006) J Neurosci 26: 1677-1687.

Membrane properties

Specific Capactiance = 1.67 $\frac{\mu F}{cm^2}$

Membrane surface area = 7.85×10^{-5} cm^2

Specific channel densities (μS/cm^2)

NaT = 24,000

NaP = 75

Kdr = 11,000

KaF = 100

KaS = 500

H-fast = 500

H-slow = 40

K-AHP = 425

K-leak = 150

Specific channel counts

NaT = 75,399

NaP = 236

Kdr = 34,558

KaF = 1,309

KaS = 2,182

H-fast = 39,270

H-slow = 5,498

K-AHP = 1,335

K-leak = 147

Single channel conductances (pS)

$\gamma_{nap} = 25$

$\gamma_{nat} = 25$

$\gamma_{kdr} = 25$

$\gamma_{kaf} = 6$

$\gamma_{kas} = 18$

$\gamma_{h} = 1$

$\gamma_{ahp} = 25$

$\gamma_{leak} = 80$

Reversal potentials (mV)

NaT = 55

NaP = 55

Kdr = -85

K-slow = -85

K-AHP = -85

H-fast = -30

H-slow = -30

K-leak = -85

State-models used for the stochastic conductances

In all cases the conducting state of the channel is indicated with an *

Transient sodium conductance

$$\begin{array}{cccccccccc} & & 3\alpha_m & & 2\alpha_m & & \alpha_m & & & \\ & m_0h_0 & \rightleftharpoons & m_1h_0 & \rightleftharpoons & m_2h_0 & \rightleftharpoons & m_3h_0^* & & \\ \alpha_h & \upharpoonleft\downharpoonright & & \upharpoonleft\downharpoonright & & \upharpoonleft\downharpoonright & & \upharpoonleft\downharpoonright & \beta_h & \\ & m_0h_1 & \rightleftharpoons & m_1h_1 & \rightleftharpoons & m_2h_1 & \rightleftharpoons & m_3h_1 & & \\ & & \beta_m & & 2\beta_m & & 3\beta_m & & & \end{array} \tag{1}$$

where,

$$\alpha_m = \frac{0.38(V_m + 33)}{1 - e^{-\frac{(V_m+33)}{9}}} \tag{2}$$

$$\beta_m = \frac{-2.3(V_m + 58)}{1 - e^{\frac{V_m+58}{12}}} \tag{3}$$

$$\alpha_h = \frac{-0.03(V_m + 48)}{1 - e^{\frac{V_m+48}{12}}} \tag{4}$$

$$\beta_h = \frac{0.05(V_m + 21)}{1 - e^{-\frac{(V_m+21)}{9}}} \tag{5}$$

Persistent sodium conductance

$$\begin{array}{cccccc} & & \alpha_m & & & \\ & m_0h_0 & \rightleftharpoons & m_1h_0^* & & \\ \alpha_h & \upharpoonleft\downharpoonright & & \upharpoonleft\downharpoonright & \beta_h & \\ & m_0h_1 & \rightleftharpoons & m_1h_1 & & \\ & & \beta_m & & & \end{array} \tag{6}$$

where,

$$\alpha_m = \frac{1.5}{1 + e^{-\frac{42.1 \quad V_m}{3}}} \tag{7}$$

$$\beta_m = \frac{1}{1 + e^{\frac{42.1 \quad V_m}{3}}} \tag{8}$$

$$\alpha_h = \frac{1.6 \times 10^{-4} \times (0.38 \times (V_m + 64.409)}{1 - e^{-0.38023(V_m+64.409)}} \tag{9}$$

$$\beta_h = \frac{1.2 \times 10^{-4} \times (-0.216 \times (V_m + 17.014))}{1 - e^{0.21598(V_m+17.014)}} \tag{10}$$

Delayed-rectifier potassium conductance

$$n_0 \underset{\beta_n}{\overset{4\alpha_n}{\rightleftharpoons}} n_1 \underset{2\beta_n}{\overset{3\alpha_n}{\rightleftharpoons}} n_2 \underset{3\beta_n}{\overset{2\alpha_n}{\rightleftharpoons}} n_3 \underset{4\beta_n}{\overset{\alpha_n}{\rightleftharpoons}} n_4^* \tag{11}$$

where,

$$\alpha_n = \frac{0.02(V_m + 38)}{1 - e^{-\frac{(V_m+38)}{10}}} \tag{12}$$

$$\beta_n = \frac{-0.018(V_m + 47)}{1 - e^{\frac{(V_m+47)}{35}}} \tag{13}$$

Fast inactivating potassium conductance (A-type)

$$\begin{array}{ccccc} & & \alpha_m & & \\ & m_0h_0 & \rightleftharpoons & m_1h_0^* & \\ \alpha_h & \upharpoonleft\downharpoonright & & \upharpoonleft\downharpoonright & \beta_h \\ & m_0h_1 & \rightleftharpoons & m_1h_1 & \\ & & \beta_m & & \end{array} \tag{14}$$

where,

$$\alpha_m = \frac{0.01(V_m + 18.3)}{1 - e^{-0.067(V_m+18.3)}} \tag{15}$$

$$\beta_m = \frac{-0.01(V_m + 18.3)}{1 - e^{0.067(V_m+18.3)}} \tag{16}$$

$$\alpha_h = \frac{-0.01(V_m + 58)}{1 - e^{0.122(V_m+58)}} \tag{17}$$

$$\beta_h = \frac{0.01(V_m + 58)}{1 - e^{-0.122(V_m+58)}} \tag{18}$$

Slowly inactivating potassium conductance (A-type)

A scaled version of the fast gating channel where activation kinetics were scaled by 0.1 and inactivation kinetics were scaled by 0.0067.

Hyperpolarization-activated, non-specific conductance ("wild-type")

$$n_0 \underset{\beta_n}{\overset{\alpha_n}{\rightleftharpoons}} n_1^* \tag{19}$$

where,

$$\alpha_n = \frac{18.3 \times 10^{-3}}{1 + e^{\frac{(V_m+114.2)}{20.33}}} \tag{20}$$

$$\beta_n = \frac{3.3 \times 10^{-2}}{1 + e^{-\frac{(V_m+51.5)}{10.94}}} \tag{21}$$

Hyperpolarization-activated, non-specific conductance ("knock-out")

$$n_0 \underset{\beta_n}{\overset{\alpha_n}{\rightleftharpoons}} n_1^* \tag{22}$$

where,

$$\alpha_n = \frac{3.6 \times 10^{-3}}{1 + e^{\frac{(V_m+148.7)}{22.45}}} \tag{23}$$

$$\beta_n = \frac{3.6 \times 10^{-3}}{1 + e^{-\frac{(V_m+50.7)}{12.49}}} \tag{24}$$

Voltage-gated AHP-like conductance

$$n_0 \underset{\beta_n}{\overset{3\alpha_n}{\rightleftharpoons}} n_1 \underset{2\beta_n}{\overset{2\alpha_n}{\rightleftharpoons}} n_2 \underset{3\beta_n}{\overset{\alpha_n}{\rightleftharpoons}} n_3^* \tag{25}$$

where,

$$\alpha_n = 1.5e^{\frac{t_{spike} \quad t}{25}} \tag{26}$$

$$\beta_n = 1.6 \tag{27}$$

Potassium conducting, voltage-independent leak conductance

$$n_0 \underset{\beta_n}{\overset{\alpha_n}{\rightleftharpoons}} n_1^* \tag{28}$$

where,

$$\alpha_n = 4 \tag{29}$$

$$\beta_n = 1 \tag{30}$$
